# Genetic analysis and physiological relationships of drought response in fennel: Interaction with mating system

**DOI:** 10.1371/journal.pone.0277926

**Published:** 2022-11-29

**Authors:** Elaheh Hosseini, Mohammad Mahdi Majidi, Fatemeh Saeidnia, Mohammad Hossein Ehtemam

**Affiliations:** 1 Department of Agronomy and Plant Breeding, College of Agriculture, Isfahan University of Technology, Isfahan, Iran; 2 Assistant Professor of Agricultural and Horticultural Science Research Department, Khorasan Razavi Agricultural and Natural Resources Research and Education Center, Agricultural Research, Education and Extension Organization, Mashhad, Iran; Central Research Institute for Dryland Agriculture, INDIA

## Abstract

The consequences of water deficit and its interaction with pollination system (deliberate selfing compared with open-pollination) on physiological, agronomic and phytochemical traits are not understood in fennel (*Foeniculum vulgare* Mill.). A research was started by creating selfed (S1) and half-sib (HS) families on a fennel germplasm in 2018. Populations were studied in the field, applying a normal and a water deficit condition during two years (2019–2020). Considerable genotypic variation was observed within S1 and HS families for all of the evaluated traits, demonstrating that selection for these traits would be successful. Consequences of water deficit were manifested as declined most of the traits; and significantly increased essential oil content, harvest index, and proline content, in both populations. Mandatory selfing reduced the performance of genotypes for most of the traits confirming the existence of inbreeding depression (ID) with higher values for plant dry weight, seed yield, essential oil content, and number of umbelets per umbel. In S1 population, some of the studied traits had higher heritability estimates under normal condition and some of them showed higher heritability under water deficit. Positive relationship between GCA and STI in OP population indicated that it is possible to identify genotypes having high values of combining ability and drought tolerance. Results of the present study suggest that physiological traits cannot be used as an indicator to distinguish drought-tolerant genotypes in S1 progenies, whereas in OP progenies Chl *a*, Chl *b*, TChl, CAR, PRO, and RWC, which had significant correlations with drought tolerance, may be used for this purpose. Based on the results contrasting genotypes were identified, which can be used to develop mapping populations for genome studies of drought tolerance and physiological traits of this species in future studies.

## Introduction

Fennel (*Foeniculum vulgare* Mill.), is a well-known medicinal and aromatic plant belonging to the Apiaceae family [1]. It is generally considered native to Southern Europe and the Mediterranean region [2], but has become widely cultivated throughout the temperate and tropical regions of the world [3]. All parts of the plant are edible and the fresh leaves and dried seeds are most commonly used as culinary ingredients. Fennel essential oil and seeds are also used to flavor prepared foods including meats, ice cream, candy, baked goods, and condiments [4]. The increasing commercial value of fennel necessitates the need to develop elite genotypes with high yield, oil content, and other desired breeding and economic traits.

Plants grow in dynamic environments where they are naturally exposed to a wide range of climatological and environmental stresses, such as water deficit or flooding, extreme temperatures, excessive light, salinity, oxidative stress, heavy metal toxicity, and nutrient deficiencies [5]. Among them, drought is particularly regarded as a major threat to ecosystems [6] and is predicted to get worse in areas exposed to severe drought, as a consequence of progressive global climate change and uncertainties in rainfall patterns. It causes deterioration of the conditions for vegetative growth, survival, and development [7, 8]; and alters morphological, physiological, biochemical, and metabolic aspects of plant species [9]. Therefore, development of drought-tolerant varieties is the ultimate mean of safeguarding the crop against the damaging effects of drought.

Plants usually respond and adapt to water deficit through changes in physiological and morphological characteristics [10]. Physiological responses can vary according to plant genotype, but in general, modifications related to water deficit include decreases in stomatal conductance and the photosynthetic efficiency and rate [11], increases in osmoprotectants such as proline and sugars [12], and reduction in the relative water content (RWC) in leaves [13]. These physiological factors may promise for characterizing drought tolerance in screening studies. Currently, efforts are directed to affordable and reliable drought tolerance and susceptibility indices based on mathematical relationships between the plant yields obtained under normal and water deficit conditions that can help in the selection of drought-tolerant genotypes [14,15]. Knowledge of the genetic association between selection indices, physiological, essential oil, and productivity traits can be useful to improve the efficiency of breeding programs.

The reproductive system of medicinal plants is investigated to provide a basis for the genetic improvement of essential oil and secondary metabolites [16]. Inbreeding depression is the most important problem which commonly occurs in outcrossing species when they are enforced to be self‐pollinated. Mating between closely related individuals is termed "inbreeding", while “inbreeding depression” refers to the relative reduction in inbred progenies’ fitness compared to outcrossed progenies [17]. These negative fitness effects are due to the higher degree of homozygosity associated with inbreeding, which increases the risks for the expression of deleterious recessive alleles [18]. However, selfing does not always have depressive effects on plants; while, in turn it facilitates the development of inbred lines, which is important for constructing special populations for genetic studies. Considering the importance of fennel as a vegetable and medicinal herb, information about the fitness under selfing and open-pollination is limited.

Half-sib (HS) matings, including polycross and open-pollination, are widely used as conventional selection methods of population improvement for estimation of combining ability, genetic variability, heritability, and other genetic parameters in quantitative genetic studies [19]. Estimation of heritability based on the analysis of HS families gives a good prediction of narrow-sense heritability; because genetic variance among HS families represents primarily the additive genetic variance contained in the phenotypic variance [20]. Moreover, the analysis of covariance between parents and offspring provides useful information concerning the inheritance of traits. The simple linear regression coefficient of offspring values on parental values is the ratio of covariance between parent and offspring, and the phenotypic variance of the parents [21].

Extremely limited information is available about the physiological response of fennel germplasm to drought stress and genetic analysis of different traits using both half-sib and selfed populations. Moreover, information is lacking on the impact of inbreeding depression (compared with open pollination), recurrent drought stresses (compared with well-watering), and their interaction with the morphological, physiological, and seed-related traits along with essential oil content of fennel genotypes. Hence, the present study aimed to (i) evaluate genetic variation of morphological, physiological traits, and essential oil content in two separate groups of fennel progeny resulting from self (S1) and open-pollination (half-sib) under two water environments, (ii) estimate the level of inbreeding depression for different traits under normal and water-deficit conditions and find genotype-specific responses in terms of inbreeding effects; (iii) assess the genetic basis of the mentioned traits through the estimating of general combining ability, heritabilities and the genetic variation of different traits using S1 and OP populations; as well as to find the changes in the genetic parameters in response to mating system and water conditions.

## Materials and methods

### Plant materials

The genetic materials of this study were consisted of 49 OP and 30 S1 families derived from open- and self-pollination of parental genotypes of fennel, respectively, during 2016 and 2017 growing seasons. The parental genotypes were mainly consisted of natural ecotypes of fennel from wide geographical areas of Iran along with foreign ecotypes kindly provided by the Leibniz Institute of Plant Genetics and Crop Plant Research (IPK) (Table 1). Parental genotypes were space-planted in the field according to a randomized complete block design with four replications in first March 2015. Every plot contained 2 rows of 4 m length of each genotype, 50 cm apart and 40 cm between plants within each row, and evaluated for four years during 2015–2018 (Table 1). During these years, irrigation was conducted with no limitation and applied when 50% of the total available water was depleted from the root zone.

**Table 1:** Information of fennel genotypes used in the study.

Code	Origin	Parental ecotype code	Parental performance		Progeny populations	
			Seed yield per plant (g)	Essential oil content (%)	Selfed (S1)	Open-pollinated (OP)
G1	Iran- Nahavand	P8	7.41	1.63	-	OP-1
G2	Germany 3	P42	26.28	2.97	S1-2	OP-2
G3	Iran- Shirvan	P9	23.33	3.35	-	OP-3
G4	Hungary 1	P38	10.42	3.82	S1-4	OP-4
G5	Iran- Karaj	P15	6.85	1.81	S1-5	OP-5
G6	Hungary 3	P51	9.12	4.14	-	OP-6
G7	Iran- Nahavand	P8	7.41	1.63	-	OP-7
G8	Unknown	P61	15.35	2.43	S1-8	OP-8
G9	Romania	P32	17.43	4.42	S1-9	OP-9
G10	Iran- Nahavand	P8	7.41	1.63	-	OP-10
G11	Iran- Varamin	P13	2.56	1.73	-	OP-11
G12	Iran- Karaj	P15	6.85	1.81	-	OP-12
G13	Switzerland	P64	22.79	1.86	S1-13	OP-13
G14	Romania	P32	17.43	4.42	-	OP-14
G15	Romania	P32	17.43	4.42	-	OP-15
G16	Iran- Nahavand	P8	7.41	1.63	-	OP-16
G17	Iran- Oroomieh	P19	26.11	3.10	-	OP-17
G18	Iran- Ebnesina	P2	21.10	2.70	-	OP-18
G19	USA 1	P33	19.40	4.91	-	OP-19
G20	Iran- Karaj	P15	6.85	1.81	-	OP-20
G21	Iran- Shirvan	P9	23.33	3.35	-	OP-21
G22	Romania	P32	17.43	4.42	-	OP-22
G23	Iran- Bushehr	P17	23.15	2.25	-	OP-23
G24	Egypt	P52	22.24	2.86	-	OP-24
G25	Germany 3	P42	26.28	2.97	-	OP-25
G26	Iran- Karaj	P15	6.85	1.81	S1-26	OP-26
G27	Iran- Isfahan	P3	30.41	2.46	S1-27	OP-27
G28	Iran- Karaj	P15	6.85	1.81	S1-28	OP-28
G29	Iran- Tabriz	P11	3.36	2.48	S1-29	-
G30	Iran- Karaj	P15	6.85	1.81	S1-30	OP-30
G31	Iran- Karaj	P15	6.85	1.81	S1-31	OP-31
G32	Iran- Kerman	P16	27.77	2.41	S1-32	OP-32
G33	Iran- Nahavand	P8	7.41	1.63	S1-33	OP-33
G34	Iran- Isfahan	P3	30.41	2.46	-	OP-34
G35	Egypt	P52	22.24	2.86	S1-35	OP-35
G36	Kazakhstan	P37	21.26	4.43	S1-36	OP-36
G37	Iran- Karaj	P15	6.85	1.81	S1-37	OP-37
G38	Iran- Tabriz	P11	3.36	2.48	S1-38	OP-38
G39	Iran- Karaj	P15	6.85	1.81	S1-39	OP-39
G40	Iran- Mashhad	P7	5.50	2.20	S1-40	OP-40
G41	Iran- Oroomieh	P19	26.11	3.10	S1-41	OP-41
G42	Iran- Ardabil	P1	16.01	2.21	S1-42	OP-42
G43	Kazakhstan	P37	21.26	4.43	S1-43	OP-43
G44	Iran- Yazd	P14	23.01	2.35	S1-44	OP-44
G45	Iran- Ardabil	P1	16.01	2.21	S1-45	OP-45
G46	Iran- Mashhad	P7	5.50	2.20	S1-46	OP-46
G47	Egypt	P52	22.24	2.86	S1-47	OP-47
G48	Iran- Nahavand	P8	7.41	1.63	S1-48	OP-48
G49	Germany 3	P42	26.28	2.97	S1-49	OP-49
G50	Hungary 3	P51	9.12	4.14	S1-50	OP-50

In 2016 and 2017, each plant of each parental genotype had half of its umbels bagged from the start of inflorescence emergence until seed harvest for obligate selfing, whereas the other half were left uncovered to enable open-pollination. At the end of the summer, seeds were separately harvested from selfed and open-pollinated umbels at full maturity stage, and then seeds of each genotype for the two harvest years were bulked to have enough seed for the field experiments. From the 50 parental ecotypes, 49 ecotypes produced enough open-pollinated (OP) seeds and 30 ecotypes produced enough selfed (S1) seeds. Therefore, two populations that are 30 S1 and 49 OP (half-sib) families were developed as the genetic material for this study (Table 1). The S1 and OP seeds were space-planted in the field in March 2018. No evaluation was done in 2018 for plant establishment. Then in 2019 and 2020 families were evaluated under normal and water deficit treatments using a split-plot experiment according to a randomized complete block design with two replications within each moisture environments (normal and water deficit). Every plot consisted of four rows of 3 m length, planted 50 cm apart, with inter-row plant distance of 40 cm.

### Experimental site

The field experiment was performed on a Typic Haplargid, silty clay loam soil at the Research Farm of Isfahan University of Technology, situated in Lavark, Najaf-Abad, Isfahan, Iran (32° 30′ N, 51° 20′ E, 1630m amsl). The soil of this area is calcareous, non-saline and non-sodic, with pH 8.3. According to Koppen classification, this region has a semi-arid and cold climate generally without rain from late May to mid-October, making irrigation necessary for growing crops. Based on 40-year meteorological data, the region’s mean annual precipitation and temperature were 140 mm and 14.5°C, respectively.

### Recurrent drought events and field evaluations

After establishment of plants during 2018, genotypes were evaluated under normal and water deficit environments during growing seasons of 2019 and 2020, in which irrigation was occurred when 50% and 85% of the total available soil water was depleted from the root zone, respectively, following accepted methods of determination of evapotranspiration [22]. Water deficit treatment was continuously applied during the growing season in each year from May 1^st^ to October 1^st^. In this period, depending on the weather conditions, the irrigation intervals were variable during the growing season and between the two moisture environments. But the amount of water that was applied for each moisture environment was identical and calculated as follows. To determine the average gravimetric soil water content and detect the irrigation times, soil moisture content was daily measured at different sites of each normal and water deficit treatment, based on standard gravimetric methods [23] at the depths of 0–20, 20–40, and 40–60 cm, using a hand auger. To determine the irrigation depth, the following formula was used:

$$I=[(\theta_{FC}‐G_{irri})/100]\times D\times B$$ (1)

where I is the irrigation depth (cm), FC is the soil gravimetric moisture percentage at the field capacity, G_irr_ is the soil gravimetric moisture percentage at the time of irrigation, D is the root-zone depth (60 cm), and B is the soil bulk density at root-zone (1.4 g cm^-3^). Water volumes that should be applied in each moisture environment were calculated by multiplication of the irrigation depth by surface area of plots. Water was delivered to the field using a drip irrigation system through a pumping station, polyethylene pipes, and drip tapes. The water volume that was applied for each treatment was measured by using a volumetric counter. Cultural practices including irrigation, fertilization, and weed control (manual) were done each year regularly.

### Measurements

Plants were allowed to establish in the field for the first year (2018) and no data were measured during this year. Twenty-three agro-morphological, physiological, and seed-related characteristics including days to germination (DG; day), days to 50% flowering (DF; day), days to 90% maturity (DM; day), plant height (PHT; cm), plant fresh weight (FW; g/plant), plant dry weight (DW; g/plant), harvest index (HI; %), number of umbels per plant (UP), number of umbelets per umbel (UU), number of seeds per umbelet (SU), seed yield per plant (SYP; g/plant), thousand seeds weight (TSW; g), seed length (SL; mm), seed width (SW; mm), essential oil content (EOC; %), relative water content (RWC; %), proline (PRO; μmol g^-1^), chlorophyll *a* content (Chl *a*; mg g^-1^); chlorophyll *b* content (Chl *b*; mg g^-1^), carotenoid content (CAR; mg g^-1^), total chlorophyll (TChl; mg g^-1^), ratio of chlorophyll *a* to chlorophyll *b* (Chl *a*/Chl *b*), and ratio of total chlorophyll to carotenoid (TChl/CAR) were evaluated under two levels of irrigation (normal and water deficit) during 2019 and 2020. Among them, phenological traits of DG, DF, and DM were measured based on the mean of each plot. Other traits were measured based on 10 randomly selected plants from the center rows of each genotype (plot) and the average values were used for analyses. HI was calculated after harvest based on the ratio of seed yield to plant dry weight multiplied by 100.

Leaf water status was determined by estimating the RWC according to Ritchie’s et al. [24] method. Measurement of chlorophyll *a* (Chl *a*), chlorophyll *b* (Chl *b*) and carotenoids (CAR) was undertaken by means of spectrophotometry, using 80% acetone as a solvent [25]. Total chlorophyll content (TChl) was calculated as TChl = Chl *a* + Chl *b*. Then the ratios of Chl *a* to Chl *b* (Chl *a* /Chl *b*) and of TChl to carotenoid (TChl/CAR) were calculated. The method which is described by the Bates et al. [26] was followed for the measurement of proline from the plants’ leaf. All of these physiological traits were measured at 50% flowering.

Inbreeding depression (ID) for each trait was calculated as follows [27]:

$$ID\;(\%)=[(M_{op}-M_{s})/M_{op}]\times 100$$ (2)

where M_op_ represents the mean value of cross-pollinated population and M_s_ represents the mean value of self-pollinated population. To differentiate the drought-tolerant and susceptible genotypes, three drought tolerance and susceptibility indices, including stress tolerance index (STI) [28], tolerance index (TOL) [29], and yield stability index (YSI) [30], were calculated for each genotype based on the seed yield under normal and water deficit conditions, as follow:

$$STI=[(Y_{pi}\times Y_{si})/{\left(Y_{mp}\right)}^{2}]$$ (3)

TOL=Ypi‐Ysi (4)

$$YSI=Y_{si}/Y_{pi}$$ (5)

where Ysi designates the seed yield of a given genotype grown under water deficit, Ypi designates that of a given genotype grown under normal condition, Yms is the seed yield mean over all genotypes grown under water deficit, and Ymp is the seed yield mean over all genotypes grown under normal condition.

### Essential oil extraction

To extract the essential oil, air-dried ripe seeds of fennel were ground to a fine powder using an electric grinder and subjected to hydrodistillation for 3 h [31] using a Clevenger-type apparatus. For each hydro-distillation run, 50 g of powdered seeds were placed in a round bottom flask. The distilled essential oil was obtained using diethyl-ether as collecting solvent (v/v), filtered, and kept in a sealed glass bottle at 4°C until use for further analyses. Essential oil content (%) was computed as follows [32]:

Essential oil content (EOC) (%) = Weight of EO recovered (g) / Weight of seed (g) × 100.

Essential oil yield (EOY) was calculated from multiplication of seed yield per plant with essential oil content for each genotype [33].

### Statistical analyses

Before analysis of variance (ANOVA), the Kolmogorov–Smirnov test was conducted to examine the normality distribution of data. The Bartlett test was used to test the homogeneity of residual variance of moisture environments. Then, to examine the differences between the genotypes, years, moisture environments, and their interactions and also to estimate the variance components, combined analysis of variance, proposed by Steel and Torrie [34], was performed using Proc MIXED of SAS release 9.4 (SAS Institute, Cary, NC, USA). As the experiment was conducted for two years in two moisture environments, a split plot in time (year) model was used for the combined analysis proposed by Nguyen and Sleper [21] and Steel and Torrie [34]. The genotype effect was considered as fixed, and year was considered random. Where the *F*-value was significant, mean comparisons of traits were undertaken using the least significant difference (LSD) test at *p* < 05 [34]. The phenotypic correlation coefficients between traits were calculated using proc CORR of SAS to determine the relationships among the studied traits. Heritability was estimated on a phenotypic mean basis averaged over replications, years, and environments according to the following formula:

$$h^{2}=\frac{\sigma_{g}^{2}}{\sigma_{g}^{2}+\frac{\sigma_{ge}^{2}}{e}+\frac{\sigma_{gy}^{2}}{y}+\frac{\sigma_{gey}^{2}}{ey}+\frac{\sigma_{\delta }^{2}}{re}+\frac{\sigma_{\epsilon }^{2}}{rey}}$$ (6)

where *h*^2^ represents the heritability, $\sigma_{g}^{2}$ is the genotype, $\sigma_{ge}^{2}$ is the genotype × environment, $\sigma_{gy}^{2}$ is the genotype × year, $\sigma_{gey}^{2}$ is the genotype × environment × year variance; $\sigma_{\delta }^{2}$ and $\sigma_{\epsilon }^{2}$ are the error variance and the residual variance, respectively; while g, e, y, and r represent the number of genotypes, environments, years, and replications, respectively [21].

Data were also subjected to ANOVA separately for normal and water deficit condition across years using a split-plot in time model with genotypes as the main plots and years as subplots. Components of variance and covariance were estimated for individual moisture environments (normal and water deficit conditions) using proc MIXED of SAS. The estimation of heritability was calculated on the basis of genotype means for normal and water deficit conditions, according to Nguyen and Sleper [21]:

$$h^{2}=\frac{\sigma_{g}^{2}}{\sigma_{g}^{2}+\frac{\sigma_{gy}^{2}}{y}+\frac{\sigma_{gr}^{2}}{r}+\frac{\sigma_{e}^{2}}{ry}}$$ (7)

where *h*^2^ is the heritability, $\sigma_{g}^{2}$ is the genotype, $\sigma_{gy}^{2}$ is the genotype × year, $\sigma_{gr}^{2}$ is the genotype × replication variance, and $\sigma_{e}^{2}$ is the error variance; while g, y, and r represent the number of genotypes, years, and replications, respectively. It should be pointed out that *h*^2^ represents an estimation of narrow-sense heritability ($h_{n}^{2}$) in the OP progenies, but an estimation of broad-sense heritability ($h_{b}^{2}$) in the selfed progenies [35].

The linear regression coefficient of OP and S1 progeny values on their parental values was calculated. In the open-pollinated populations, the linear regression coefficient is based on offspring and one parent; then, the linear regression coefficient of OP values on their parental values was multiplied by two to obtain an estimate of narrow-sense heritability [36]. In selfed populations (S1), the covariance between parents and offspring includes dominance and dominance-type epistasis in addition to additive and additive-type epistasis. Regression of S1 progeny on the parental plants thus provides an estimate of broad-sense heritability [37]. The level of genetic variation was estimated with the calculation of the genotypic coefficient of variation (GCV) as follows:

$$GCV=(\frac{\sigma_{g}}{\mu })\times 100$$ (8)

where *σ*_*g*_ is the standard deviation of the genotypic variance, and μ is the phenotypic mean [36].

General combining ability (GCA) was calculated for important traits on the combined data of two moisture environments as the deviation of each HS family from the population mean as defined by Wricke and Weber [38]. Moreover, to evaluate the relationships among the fennel populations and all of the measured traits, principal component analysis (PCA) was done based on a correlation matrix on all evaluated traits using Statgraphics statistical software 17.2 (Statgraphics Technologies, The Plains, VA, USA).

### Results

#### Analysis of variance, mean comparisons, and genetic variation

Results from combined analysis of variance for both populations indicated that there were significant differences (P < 0.05) between the normal and water deficit conditions for all traits except for Chl *a*/Chl *b* and TChl/CAR in S1 population, and DG, PRO, and Chl *a*/Chl *b* in OP one. The effect of genotype was significant for all traits, indicating significant variation among the selected genotypes with a broad range for each attribute. The interactions between genotype and moisture environment were also significant for all the measured traits except for DF and UU in S1 population, and DG and DF in OP population (S1 and S2 Tables).

Mean comparisons of S1 and OP populations for important traits based on the average of two years are given in S3 and S4 Tables, respectively. In S1 population, under normal condition, days to 50% flowering (DF) ranged from 46.00 to 71.75 days with an average of 56.75 days. Among the studied families, S1-48, S1-42, and S1-4 were the early flowering families, while S1-29 was the late flowering one. Under water deficit condition, it ranged from 42.00 to 69.00 days with an average of 53.61 days. Families, S1-48, S1-4, and S1-42, were the early flowering families, while S1-29 was the latest flowering (S3 Table). Seed yield per plant (SYP) varied considerably and ranged from 1.47 to 18.53 g. The highest value of this trait was obtained for S1-9 and the lower values were detected for S1-42 and S1-48. Under water deficit, its range was from 0.72 g to 8.13 g. Family S1-9 produced the highest seed yield, and families S1-43, S1-40, S1-42, S1-48, S1-46, S1-44, and S1-5 produced lower values of seed yield (S3 Table). The lower values of thousand seed weight (TSW) under normal condition were obtained for S1-49 and S1-44, and the higher values were detected for S1-29 and S1-35, respectively. However, under water deficit condition, the lower values of this trait were obtained for S1-9, S1-49, S1-41, S1-40, and S1-13; and the higher values of it were detected for S1-47, S1-45, S1-48, S1-37, and S1-27, respectively (S3 Table). Families S1-47 and S1-33 under normal condition, and S1-36 and S1-42 under water deficit condition showed higher values of essential oil content (EOC), respectively. Meanwhile, under normal condition S1-8, and under water deficit S1-43, S1-30, S1-5, and S1-28 had the lower values of this trait (S3 Table).

In OP population, under normal condition, DF ranged from 51.00 to 75.00 days with an average of 65.03 days. Among the studied families, OP-14, OP-15, and OP-6 were the early flowering families, while OP-49, OP-33, OP-3, OP-34, OP-35, OP-41, OP-40, OP-43, and OP-23 were late flowering ones. Under water deficit condition, it ranged from 48.75 to 72.50 days with an average of 61.18 days. Families, OP-14, OP-15, OP-4, and OP-6 were the early flowering families, while OP-33, OP-49, OP-3, OP-41, and OP-35 were late flowering (S4 Table). Seed yield ranged from 16.85 to 64.14 g. The higher values of this trait were obtained for OP-9 and OP-38, and the lower values were detected for OP-24, OP-6, OP-1, OP-15, OP-36, OP-2, and OP-43, respectively (S4 Table). The parental genotypes of families OP-9 and OP-38 had the higher GCA for SYP and therefore are good combiners. While, the parental genotypes of families OP-24, OP-6, OP-1, OP-15, OP-36, OP-2, and OP-43 showed lower values of GCA for SYP and therefore are bad combiners (S5 Table). Under water deficit, the range of SYP was from 3.09 g to 36.35 g. Family OP-38 produced the highest SYP, and families OP-15, OP-6, OP-16, OP-1, OP-10, and OP-24 produced lower values of it, respectively (S4 Table). In this condition, the parental genotype of OP-8 had also the highest GCA and is a good combiner for this trait. Meanwhile, the parental genotypes of OP-15, OP-6, OP-16, OP-1, OP-10, and OP-24 had lower values of GCA for SYP and hence are bad combiners for this trait (S5 Table). The lowest value of TSW under normal condition was obtained for OP-6, and the higher values were detected for OP-33 and OP-35, respectively (S4 Table). The higher GCAs of this trait belonged to the parental genotypes of OP-33 and OP-35. Therefore, these genotypes are good combiners for TSW. Parental genotype OP-6 had the lowest GCA for TSW and therefore, is not a good combiner (S5 Table). However, under water deficit, the lowest value of this trait was obtained for OP-6; and the higher values of it were detected for OP-33, OP-35, and OP-31, respectively (S4 Table). The lowest GCA of TSW in this condition belonged to the parental genotype OP-6. Therefore, this genotype is not a good combiner for this trait. Parental genotypes OP-33, OP-35, and OP-31 had high GCAs for TSW and therefore, are good combiners (S5 Table). Families OP-33, OP-36, OP-42, OP-44, and OP-47 under normal condition and OP-11 under water deficit showed the higher values of EOC, respectively (S4 Table). The parental genotypes of these families had also higher GCAs and are good combiners (S5 Table). Meanwhile, OP-6 from Hungary showed the lowest EOC under both normal and water deficit conditions (S4 Table), and its parental genotype had the lowest GCA for EOC.

Estimates of genetic coefficient of variation (GCV) for S1 and OP populations under normal and water deficit conditions are given in Table 2. In selfed progenies, relatively high GCV was observed for plant dry weight (DW), harvest index (HI), Chlorophyll *b* (Chl *b*), and the ratio of chlorophyll *a* to chlorophyll *b* (Chl *a*/Chl *b*) under normal condition, and for plant fresh weight (FW), DW, HI, Chl *b*, CAR, Chl *a*/Chl *b*, and the ratio of total chlorophyll to carotenoid (TChl/CAR) under water deficit. However, for the other evaluated traits moderate to low variations were observed. In open-pollinated progenies, GCV was relatively high for FW, DW, HI, and Chl *a*/Chl *b* under normal condition, and for days to germination (DG), FW, DW, HI, and Chl *a*/Chl *b* under water deficit condition. In contrast, variation for the remaining studied traits was moderate to low (Table 2).

**Table 2:** The effect of water deficit, range, and genotypic coefficient of variation (GCV) of different traits recorded under normal and water deficit conditions in fennel genotypes during two years (2019–2020).

Traits	Selfed population (S1)			Open-pollinated population (OP)			GCV			
							S1		OP	
	Normal	Stress	Change (%)	Normal	Stress	Change (%)	Normal	Stress	Normal	Stress
DG (day)	4.825 ^a^	4.100 ^a^	-15.11	5.944 ^b^	6.801 ^a^	14.48	11.91	17.51	18.38	20.56
DF (day)	56.750 ^a^	53.608 ^b^	-5.53	65.031 ^a^	61.179 ^b^	-5.92	4.35	5.65	3.87	4.30
DM (day)	123.025^a^	101.800^b^	-17.26	133.485^a^	114.061^b^	-14.55	2.93	4.54	2.08	2.86
PHT (cm)	59.338 ^a^	46.150 ^b^	-22.23	81.640 ^a^	60.598 ^b^	-25.77	7.01	10.58	9.52	10.10
FW (g/plant)	133.694^a^	61.855^b^	-53.73	932.920^a^	142.026^b^	-84.77	17.95	22.54	19.76	33.02
DW (g/plant)	37.604 ^a^	10.408 ^b^	-72.31	109.431^a^	27.627 ^b^	-74.75	22.54	27.43	37.90	42.40
UP	14.083 ^a^	7.477 ^b^	-46.87	27.650 ^a^	15.058 ^b^	-45.53	13.92	18.25	11.26	17.51
UU	12.860 ^a^	11.019 ^b^	-14.31	16.543 ^a^	12.575 ^b^	-23.94	5.41	7.86	5.99	5.86
SU	21.552 ^a^	16.718 ^b^	-22.41	29.954 ^a^	20.280 ^b^	-32.29	8.71	7.15	6.14	6.80
SYP (g/plant)	8.753 ^a^	3.432 ^b^	-60.80	31.280 ^a^	13.604 ^b^	-56.52	15.15	13.70	12.38	13.75
HI (%)	20.735 ^b^	27.415 ^a^	32.16	29.680 ^b^	38.102 ^a^	28.37	21.45	25.04	25.14	19.48
TSW (g)	2.626 ^a^	1.984 ^b^	-24.71	3.935 ^a^	3.112 ^b^	-20.86	5.10	7.31	7.00	9.06
EOC (%)	1.339 ^b^	1.711 ^a^	27.61	2.387 ^b^	3.227 ^a^	35.15	11.81	8.29	10.12	6.79
SL (mm)	4.236 ^a^	3.501 ^b^	-17.45	6.004 ^a^	5.060 ^b^	-15.67	4.57	7.08	5.32	5.85
SW (mm)	1.418 ^a^	1.228 ^b^	-13.38	2.129 ^a^	1.833 ^b^	-14.08	3.20	4.15	3.87	4.49
RWC (%)	70.386 ^a^	62.475 ^b^	-11.25	75.485 ^a^	66.666 ^b^	-11.68	2.31	2.95	2.75	3.06
PRO (μmol g^-1^)	0.034 ^b^	0.090 ^a^	200.00	0.028 ^b^	0.042 ^a^	33.33	16.61	14.83	16.17	12.40
Chl *a* (mg g ^-1^)	2.230 ^a^	1.192 ^b^	-46.64	3.066 ^a^	1.667 ^b^	-45.60	12.88	15.25	11.71	16.99
Chl *b* (mg g ^-1^)	0.394 ^a^	0.120 ^b^	-69.23	0.638 ^a^	0.286 ^b^	-54.69	28.56	32.30	17.70	18.20
CAR (mg g ^-1^)	0.516 ^b^	1.048 ^a^	50.48	0.798 ^b^	1.294 ^a^	37.98	11.62	21.55	9.75	14.49
TChl (mg g ^-1^)	2.624 ^a^	1.313 ^b^	-50.00	3.704 ^a^	1.953 ^b^	-47.30	13.58	16.27	10.69	15.13
Chl *a*/Chl *b*	7.715 ^a^	17.192 ^a^	122.67	5.880 ^a^	7.241 ^a^	23.13	26.61	49.79	20.43	31.10
TChl/CAR	2.551 ^b^	9.814 ^a^	284.71	2.917 ^a^	2.529 ^a^	-13.36	13.46	186.03	8.88	14.25

“a” and “b” show the comparison between normal and water deficit conditions for each trait. Means followed by the same letters in each trait and each population are not significantly different according to the LSD test at the 5% level of probability.

CAR, Carotenoid content; Chl *a*, Chlorophyll *a* content; Chl *b*, Chlorophyll *b* content; Chl *a*/Chl *b*, Ratio of chlorophyll *a* to chlorophyll *b* content; DF, Days to flowering; DG, Days to germination; DM, Days to maturity; DW, Plant dry weight; EOC, Essential oil content; FW, Plant fresh weight; HI, Harvest index; PHT, Plant height; PRO, Proline content; RWC, Relative water content; SL, Seed length; SU, Number of seeds per umbelets; SW, Seed width; SYP, Seed yield per plant; TChl, Total chlorophyll content; TChl/CAR, Ratio of total chlorophyll to carotenoid content; TSW, Thousand seed weight; UP, Number of umbels per plant; UU, Number of umbelets per umbel.

### Effect of deficit irrigation and mating system

On average, selfing reduced the magnitude of mean performance of all traits except for DG, PHT, UU, SU, PRO, Chl *a*, CAR, Chl *a*/Chl *b*, and TChl/CAR compared with open-pollination, (Table 3), indicating evidence for inbreeding depression. Under normal condition, DG, PHT, CAR, and TChl/CAR were decreased in S1 population compared with OP one; while, under water deficit, there was no significant difference between S1 and OP populations in terms of these traits. In contrast, under water deficit, Chl *b* was decreased in S1 population compared with OP one; while this was not the case under normal condition. However, self-pollination did not affect the proline content of studied germplasm.

**Table 3:** Effect of selfing on different traits of fennel under normal and water deficit conditions during two years (2019–2020).

Traits	Normal environment			Water stress environment		
	S1	OP	Change (%)	S1	OP	Change (%)
DG (day)	4.825^b^	5.944 ^a^	-18.69	4.100 ^a^	6.801 ^a^	-39.71
DF (day)	56.750 ^b^	65.031 ^a^	-12.73	53.608 ^b^	61.179 ^a^	-12.37
DM (day)	123.025 ^b^	133.485 ^a^	-7.83	101.800 ^b^	114.061 ^a^	-10.75
PHT (cm)	59.338 ^b^	81.640 ^a^	-27.32	46.150 ^a^	60.598 ^a^	-23.84
FW (g/plant)	133.694 ^b^	932.920 ^a^	-85.67	61.855 ^b^	142.026 ^a^	-56.45
DW (g/plant)	37.604 ^b^	109.431 ^a^	-65.64	10.408 ^b^	27.627 ^a^	-62.32
UP	14.083 ^b^	27.650 ^a^	-49.08	7.477 ^b^	15.058 ^a^	-50.33
UU	12.860 ^a^	16.543 ^a^	-22.25	11.019 ^a^	12.575 ^a^	-12.40
SU	21.552 ^a^	29.954 ^a^	-28.05	16.718 ^a^	20.280 ^a^	-17.55
SYP (g/plant)	8.753 ^b^	31.280 ^a^	-72.03	3.432 ^b^	13.604 ^a^	-74.78
HI (%)	20.735 ^b^	29.680 ^a^	-30.12	27.415 ^b^	38.102 ^a^	-28.06
TSW (g)	2.626 ^b^	3.935 ^a^	-33.08	1.984 ^b^	3.112 ^a^	-36.33
EOC (%)	1.339 ^b^	2.387 ^a^	-43.93	1.711 ^b^	3.227 ^a^	-47.06
SL (mm)	4.236 ^b^	6.004 ^a^	-29.33	3.501 ^b^	5.060 ^a^	-30.83
SW (mm)	1.418 ^b^	2.129 ^a^	-33.33	1.228 ^b^	1.833 ^a^	-32.79
RWC (%)	70.386 ^b^	75.485 ^a^	-6.76	62.475 ^b^	66.666 ^a^	-6.30
PRO (μmol g^-1^)	0.034 ^a^	0.028 ^a^	0.00	0.090 ^a^	0.042 ^a^	125.00
Chl *a* (mg g ^-1^)	2.230 ^b^	3.066 ^a^	-27.36	1.192 ^b^	1.667 ^a^	-28.74
Chl *b* (mg g ^-1^)	0.394 ^a^	0.638 ^a^	-39.06	0.120 ^b^	0.286 ^a^	-58.62
CAR (mg g ^-1^)	1.048 ^b^	1.294 ^a^	-18.60	0.516 ^a^	0.798 ^a^	-35.00
TChl (mg g ^-1^)	2.624 ^b^	3.704 ^a^	-29.19	1.313 ^b^	1.953 ^a^	-32.82
Chl *a*/Chl *b*	7.715 ^a^	5.880 ^a^	31.29	17.192 ^a^	7.241 ^a^	137.43
TChl/CAR	2.551 ^b^	2.917 ^a^	-12.67	9.814 ^a^	2.529 ^a^	287.75

“a” and “b” show the comparison between S1 and OP populations for each trait. Means followed by the same letters in each trait and each moisture environment are not significantly different according to the LSD test at the 5% level of probability.

CAR, Carotenoid content; Chl *a*, Chlorophyll *a* content; Chl *b*, Chlorophyll *b* content; Chl *a*/Chl *b*, Ratio of chlorophyll *a* to chlorophyll *b* content; DF, Days to flowering; DG, Days to germination; DM, Days to maturity; DW, Plant dry weight; EOC, Essential oil content; FW, Plant fresh weight; HI, Harvest index; PHT, Plant height; PRO, Proline content; RWC, Relative water content; SL, Seed length; SU, Number of seeds per umbelets; SW, Seed width; SYP, Seed yield per plant; TChl, Total chlorophyll content; TChl/CAR, Ratio of total chlorophyll to carotenoid content; TSW, Thousand seed weight; UP, Number of umbels per plant; UU, Number of umbelets per umbel.

Moreover, genotype-specific response was observed for inbreeding depression (ID) within the germplasm for different traits. Under normal condition, the highest and lowest ID values for SYP were observed in genotypes 42 and 31 and under water deficit were detected in genotypes 40 and 9, respectively. For EOC, the highest value of ID was observed for genotypes 8 and 43 under normal and water deficit, respectively; while the lowest value of this parameter was detected for genotypes 30 and 36 under normal and water deficit conditions, respectively. The highest and lowest value of ID for TSW was observed in genotypes 33 and 8 under normal condition, and in genotypes 33 and 48 under water deficit, respectively. For RWC, the highest and lowest values of ID were detected in genotypes 48 and 40 under normal condition, respectively; while under water deficit these values were obtained in genotypes 49 and 45 (Table 4).

**Table 4:** The percentage of inbreeding depression in some important traits of selfed fennel genotypes (S1) under normal and water deficit conditions during two years (2019–2020).

Code	Days to flowering (day)		Plant height (cm)		Plant dry weight (g/plant)		Seed yield per plant (g/plant)		Essential oil content (%)		Harvest index (%)	
	Normal	Stress	Normal	Stress	Normal	Stress	Normal	Stress	Normal	Stress	Normal	Stress
G2	-3.34	-1.57	-17.37	-24.27	-59.58	-63.94	-73.07	-66.74	-70.62	-52.90	-24.27	1.36
G4	-17.01	-13.62	-18.99	-16.52	-68.17	-57.45	-61.35	-77.29	-52.77	-54.46	10.18	-18.76
G5	-6.57	-11.68	-17.67	-20.98	-41.61	-55.03	-64.57	-89.56	-56.71	-58.60	-23.58	-40.50
G8	-9.04	-0.77	-15.18	-7.24	-41.29	-43.20	-69.13	-75.13	-75.67	-59.06	-24.84	-21.83
G9	-3.65	-3.52	-35.54	-18.00	-69.69	-56.97	-71.08	-51.54	-66.76	-50.65	-3.30	13.62
G13	-6.18	-1.48	-19.86	-20.35	-79.44	-65.31	-63.35	-63.83	-42.39	-51.91	65.57	74.85
G26	-28.41	-27.91	-14.21	-21.37	-68.29	-53.09	-78.28	-88.58	-40.68	-42.39	-10.05	-61.40
G27	-19.28	-21.10	-28.99	-29.67	-44.72	-70.69	-78.92	-73.20	-37.17	-38.21	-26.47	10.50
G28	-16.96	-19.78	-14.66	-26.50	-34.13	-23.58	-63.63	-72.41	-57.19	-64.83	-35.60	-47.77
G30	-5.27	-4.74	-27.45	-33.67	-59.84	-80.13	-59.69	-72.02	-24.36	-53.38	25.48	27.28
G31	-22.01	-26.08	-21.01	-32.76	-81.99	-74.44	-55.17	-67.91	-45.10	-41.22	119.03	34.07
G32	-18.55	-21.35	-33.83	-35.77	-91.37	-55.44	-79.77	-89.80	-38.91	-55.13	112.95	-54.84
G33	-19.87	-23.64	-36.82	-37.89	-55.71	-79.04	-65.23	-70.42	-44.40	-57.61	-3.00	24.51
G35	-18.01	-20.70	-32.47	-43.30	-77.75	-80.65	-61.93	-68.02	-29.76	-46.74	51.41	36.18
G36	-16.26	-16.35	-43.97	-23.31	-30.02	-38.82	-63.50	-63.93	-57.87	-34.55	-37.98	-30.78
G37	-7.16	0.17	-12.05	-12.83	-78.76	-74.43	-85.44	-82.60	-56.26	-43.04	-24.62	-30.21
G38	-18.22	-13.53	-20.92	-34.28	-73.67	-87.46	-73.12	-81.37	-58.68	-58.25	15.27	27.34
G39	-16.66	-17.19	-28.22	-22.39	-78.46	-68.92	-70.25	-89.82	-47.87	-44.61	24.57	-57.09
G40	-18.96	-16.50	-17.13	-17.10	-80.61	-77.97	-85.69	-95.27	-57.04	-44.73	-20.14	-62.65
G41	-15.87	-17.76	-30.16	-34.13	-55.17	-81.61	-81.28	-84.73	-49.91	-60.14	-44.68	-12.89
G42	-29.71	-32.47	-20.53	-39.26	-74.22	-37.93	-95.74	-93.97	-55.65	-40.42	-77.30	-80.77
G43	-26.85	-21.38	-43.13	-23.55	-74.12	-77.14	-84.25	-92.73	-42.94	-69.64	-35.30	-60.24
G44	-16.18	-11.89	-33.75	-27.02	-66.82	-50.62	-74.44	-91.97	-48.94	-43.69	-17.92	-66.93
G45	-10.24	-8.91	-19.37	-21.63	-87.98	-55.27	-70.93	-73.38	-50.48	-50.24	134.30	-34.82
G46	-9.15	-8.12	-28.72	-20.95	-60.13	-89.34	-84.55	-89.82	-29.95	-53.50	-57.25	-1.46
G47	-27.34	-25.29	-40.45	-33.88	-72.06	-35.81	-83.58	-80.77	-39.67	-36.54	-35.56	-53.46
G48	-26.50	-27.97	-39.82	-44.94	-70.88	-53.45	-91.35	-93.55	-35.42	-44.98	-56.01	-66.03
G49	-11.14	-10.28	-29.04	-17.00	-55.91	-66.07	-73.68	-82.46	-40.95	-42.56	-35.93	-36.94
G50	-9.19	-9.56	-19.03	-12.01	-68.18	-75.39	-64.68	-69.69	-25.83	-40.07	20.40	27.37
Mean	-15.64	-15.00	-26.22	-25.95	-65.54	-63.08	-73.37	-79.05	-47.59	-49.45	-0.51	-19.39
LSD	4.69	5.06	7.80	11.20	13.17	14.58	7.16	7.25	7.29	6.81	30.52	27.11
Code	Number of umbels per plant		Number of umbelets per umbel		Number of seeds per umbelets		Seed length (mm)		Seed width (mm)		Thousand seed weight (g)	
	Normal	Stress	Normal	Stress	Normal	Stress	Normal	Stress	Normal	Stress	Normal	Stress
G2	-53.45	-44.36	-12.05	-16.69	-33.77	-9.06	-35.87	-34.98	-24.93	-32.07	-39.40	-44.46
G4	-46.86	-62.42	-32.59	-18.22	-18.18	5.02	-44.25	-35.93	-33.40	-41.86	-29.62	-38.34
G5	-24.39	-57.37	-26.32	-6.62	-28.43	-17.53	-30.91	-24.57	-26.48	-31.34	-30.59	-46.66
G8	-31.23	-17.01	-25.54	-11.97	-41.62	-21.42	-31.39	-40.73	-34.51	-35.38	-25.67	-35.30
G9	-35.11	-38.05	-34.81	-25.10	-23.36	-25.05	-21.07	-31.67	-29.64	-30.16	-27.99	-37.71
G13	-39.56	-50.04	-40.61	-21.77	-12.92	-23.76	-26.21	-31.00	-35.03	-36.31	-36.94	-49.81
G26	-48.30	-65.14	-23.16	0.66	-37.44	-9.72	-24.92	-30.61	-34.71	-35.40	-45.65	-51.70
G27	-54.27	-36.88	-16.99	0.04	-30.45	-7.75	-29.08	-32.32	-33.65	-36.48	-33.65	-33.05
G28	-30.34	-40.44	-26.68	3.19	-35.35	-14.87	-28.20	-28.65	-27.89	-34.20	-39.58	-39.72
G30	-34.83	-54.40	-31.00	-3.46	-33.87	-23.85	-30.67	-54.82	-29.05	-36.54	-26.76	-35.67
G31	-39.35	-43.39	-29.02	-18.62	-36.93	-20.44	-24.20	-39.63	-33.37	-36.17	-39.45	-49.21
G32	-81.48	-66.62	-12.17	-15.02	-30.55	-25.08	-32.27	-24.30	-32.80	-33.30	-29.56	-36.50
G33	-53.15	-61.57	-35.39	-43.59	-42.90	-6.41	-37.01	-32.10	-41.44	-37.66	-46.18	-53.11
G35	-47.39	-42.28	-36.12	-15.84	-22.32	2.78	-36.57	-52.38	-32.62	-46.46	-34.73	-42.49
G36	-41.71	-29.44	-29.52	-11.80	-26.68	1.29	-26.97	-38.40	-38.54	-42.34	-33.88	-36.55
G37	-56.77	-57.54	-15.63	15.59	-29.01	-14.12	-30.68	-28.92	-34.48	-31.57	-32.07	-34.65
G38	-63.19	-34.01	-26.46	-6.42	-29.95	-1.93	-26.42	-29.86	-33.96	-32.81	-31.18	-43.35
G39	-55.12	-66.54	-33.60	-31.27	-34.88	-14.10	-34.60	-30.22	-32.25	-31.49	-37.48	-37.91
G40	-66.27	-79.74	-17.01	-18.07	-33.13	-12.92	-31.18	-27.62	-29.15	-32.92	-42.36	-47.82
G41	-41.31	-36.58	-18.65	-11.15	-21.41	-9.69	-34.04	-30.77	-35.40	-37.72	-36.63	-53.11
G42	-65.44	-74.89	-33.90	-12.78	-29.46	-17.00	-34.62	-33.26	-32.86	-28.96	-41.07	-28.59
G43	-53.89	-78.83	-16.17	-12.93	-44.86	-16.76	-27.42	-26.39	-31.94	-35.58	-27.22	-44.13
G44	-53.48	-59.76	-27.86	-21.08	-33.61	-16.95	-38.05	-35.78	-35.41	-42.63	-39.16	-37.08
G45	-47.08	-49.44	-28.64	-26.87	-12.63	-9.06	-31.20	-31.74	-29.32	-31.99	-35.06	-29.99
G46	-46.30	-58.22	-3.85	-16.65	-37.44	-13.54	-26.43	-37.04	-30.91	-35.67	-41.18	-41.24
G47	-67.67	-81.27	-26.53	-17.56	-37.70	-24.76	-35.01	-26.71	-28.64	-40.78	-35.45	-31.89
G48	-54.64	-71.66	-27.37	-22.81	-25.50	-15.95	-35.79	-47.18	-28.07	-28.94	-30.98	-27.30
G49	-55.12	-63.87	-27.52	-17.65	-21.54	-4.08	-27.69	-26.87	-37.79	-46.17	-43.34	-44.19
G50	-58.86	-78.07	-29.18	-19.94	-42.71	-21.48	-35.44	-38.77	-32.06	-34.35	-36.58	-34.69
Mean	-49.88	-55.17	-25.67	-14.63	-30.64	-13.39	-31.32	-33.90	-32.42	-35.77	-35.50	-40.21
LSD	11.26	13.51	14.20	19.55	13.10	24.47	5.59	5.29	4.13	3.67	6.27	6.46
Code	Relative water content (%)		Proline content (μmol g^-1^)		Chlorophyll *a* content (mg g ^-1^)		Chlorophyll *b* content (mg g ^-1^)		Carotenoid content (mg g ^-1^)		Total chlorophyll content (mg g ^-1^)	
	Normal	Stress	Normal	Stress	Normal	Stress	Normal	Stress	Normal	Stress	Normal	Stress
G2	-5.40	-5.25	82.56	191.55	-12.64	-34.31	-37.62	-79.77	-8.19	-33.18	-18.61	-37.61
G4	-8.50	-16.61	88.22	341.41	-22.77	-54.92	-43.00	-57.43	-9.98	-31.53	-26.13	-55.24
G5	-2.24	-9.79	11.03	233.80	-28.76	-58.44	-49.66	-67.00	-21.70	-22.17	-32.86	-59.55
G8	-10.11	-12.31	91.96	259.94	-47.97	-64.58	-48.99	-46.97	-36.58	-36.15	-48.37	-63.13
G9	-4.20	-4.37	268.01	157.45	-80.70	-81.41	-48.48	-86.90	-66.42	-95.82	-75.25	-81.58
G13	-4.53	-12.69	103.33	100.52	-23.36	-41.33	-39.32	-65.18	-31.70	-64.53	-26.53	-45.55
G26	-4.44	-5.08	1.26	172.19	-60.39	-28.41	-72.63	-81.34	-28.62	-57.54	-64.62	-35.61
G27	-2.00	-4.13	71.95	69.88	-14.25	-15.78	-38.68	-54.53	-3.80	-17.76	-17.46	-20.29
G28	-9.46	-13.51	54.67	139.27	-14.54	-26.70	-54.59	-69.83	-21.11	-69.61	-23.89	-33.97
G30	-7.76	-10.69	-44.11	129.22	-15.93	-49.48	-46.90	-72.87	-7.89	-63.14	-20.87	-53.33
G31	-7.65	-2.67	98.47	253.38	-37.46	-21.79	-26.71	-52.38	-23.35	-20.95	-36.24	-25.80
G32	-3.50	-14.00	-10.81	52.11	-28.94	-6.73	-37.99	-74.43	-18.64	-11.47	-31.93	-19.88
G33	-4.99	-7.30	-28.19	97.56	-21.64	-14.99	-54.23	-27.77	-27.72	-23.86	-30.65	-17.07
G35	-7.09	-10.65	86.33	197.79	-30.50	-28.65	-49.66	-42.07	-29.37	-34.81	-34.09	-31.83
G36	-9.88	-12.19	-63.48	17.59	-5.59	-27.33	-38.32	-51.23	-4.48	-14.81	-10.34	-31.19
G37	-7.57	-6.10	3.90	182.11	-38.87	-27.81	-25.97	-24.32	-33.11	-29.45	-38.11	-27.53
G38	-7.06	-10.76	-4.35	134.00	-22.45	-20.12	-47.37	-25.52	-24.40	-24.11	-28.30	-21.09
G39	-7.02	-2.14	-12.78	90.96	-11.87	-29.22	-23.30	-8.58	-42.62	-82.64	-14.65	-26.08
G40	-1.29	-5.44	-32.75	13.56	-10.38	-38.73	-60.30	-39.02	-0.16	-45.62	-22.76	-43.05
G41	-12.48	-8.69	86.28	140.08	-25.31	-22.32	-20.11	-83.80	-18.93	-21.80	-24.75	-29.79
G42	-6.44	-3.23	143.85	279.62	-15.71	-31.38	-43.80	-66.02	-11.01	-34.29	-21.21	-36.95
G43	-7.62	-6.13	23.37	186.30	-13.27	-61.93	-35.43	-59.67	-6.91	-48.69	-17.86	-61.58
G44	-14.91	-7.35	3.42	211.17	-41.62	-27.72	-44.85	-67.18	-34.03	-12.91	-42.14	-32.11
G45	-3.24	-0.64	-1.54	155.94	-25.12	-18.68	-32.64	-87.15	-7.02	-20.31	-27.40	-27.23
G46	-4.23	-3.22	80.32	108.43	-37.28	-27.69	-44.44	-68.06	-24.44	-55.10	-39.24	-38.17
G47	-2.39	-13.64	-15.06	166.31	-40.28	-47.09	-47.30	-77.27	-22.27	-41.40	-41.51	-52.37
G48	-16.59	-6.14	146.04	213.97	-34.45	-28.44	-22.79	-87.96	-23.25	-53.60	-32.06	-40.67
G49	-9.05	-17.52	-46.64	28.43	-20.49	-35.17	-40.34	-54.33	-8.86	-23.38	-24.76	-38.00
G50	-9.20	-8.19	134.12	85.20	-13.42	-63.09	-50.54	1.36	-2.16	-1.55	-18.41	-60.65
Mean	-6.93	-8.29	45.50	152.06	-27.45	-35.66	-42.27	-57.84	-20.65	-37.66	-30.72	-39.55
LSD	4.45	3.83	63.67	87.66	15.67	19.92	25.17	30.10	17.07	23.07	14.00	15.97

LSD, least significant difference.

**Fig 1:**
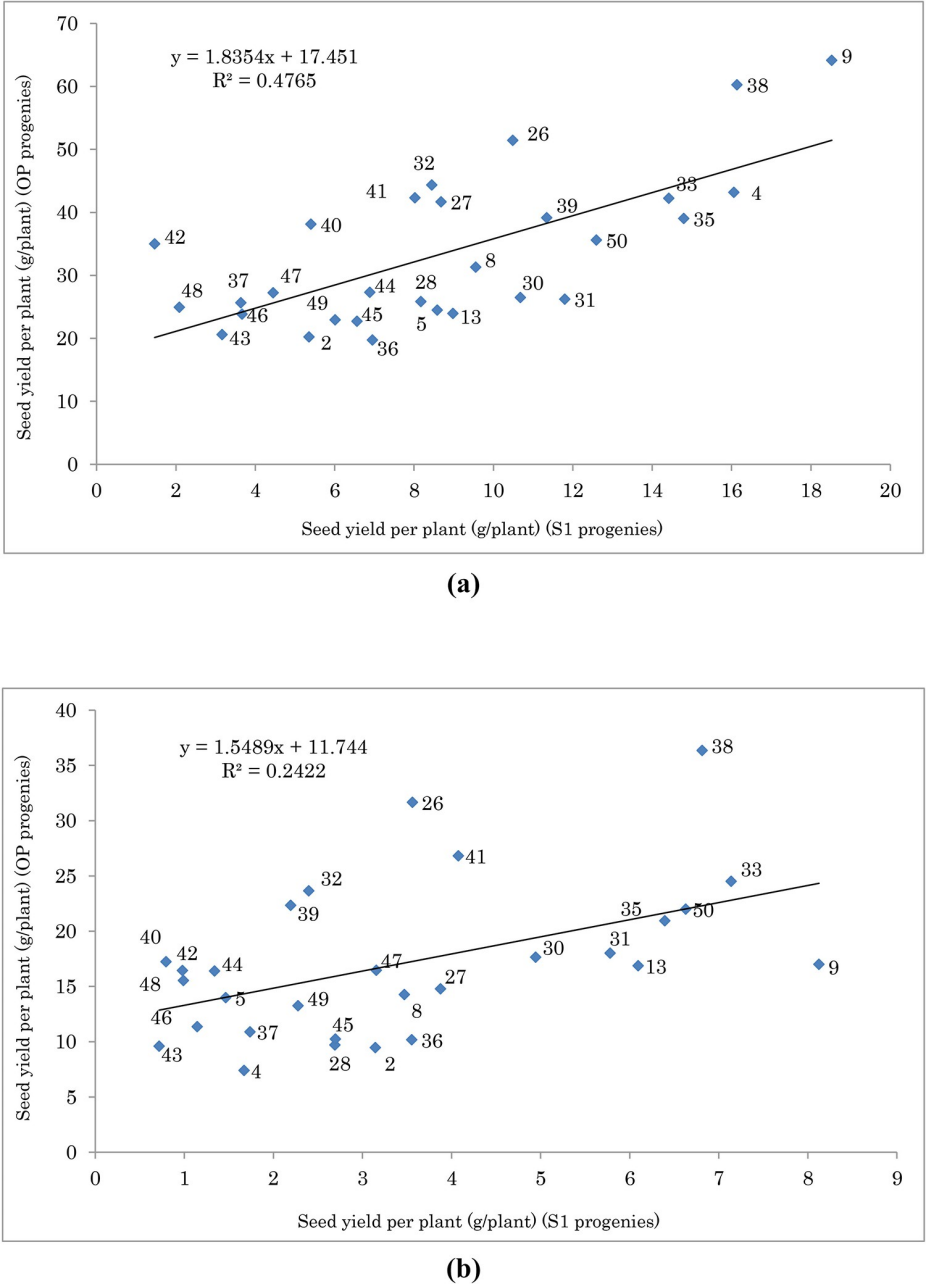
Seed yield per plant (SYP) of 29 selfed (S1) and open-pollinated (OP) populations of fennel under normal (a) and water deficit (b) conditions. The F-test indicated that the model is significant for normal condition in 0.01 probability level, and for water deficit condition is not significant.

**Fig 2:**
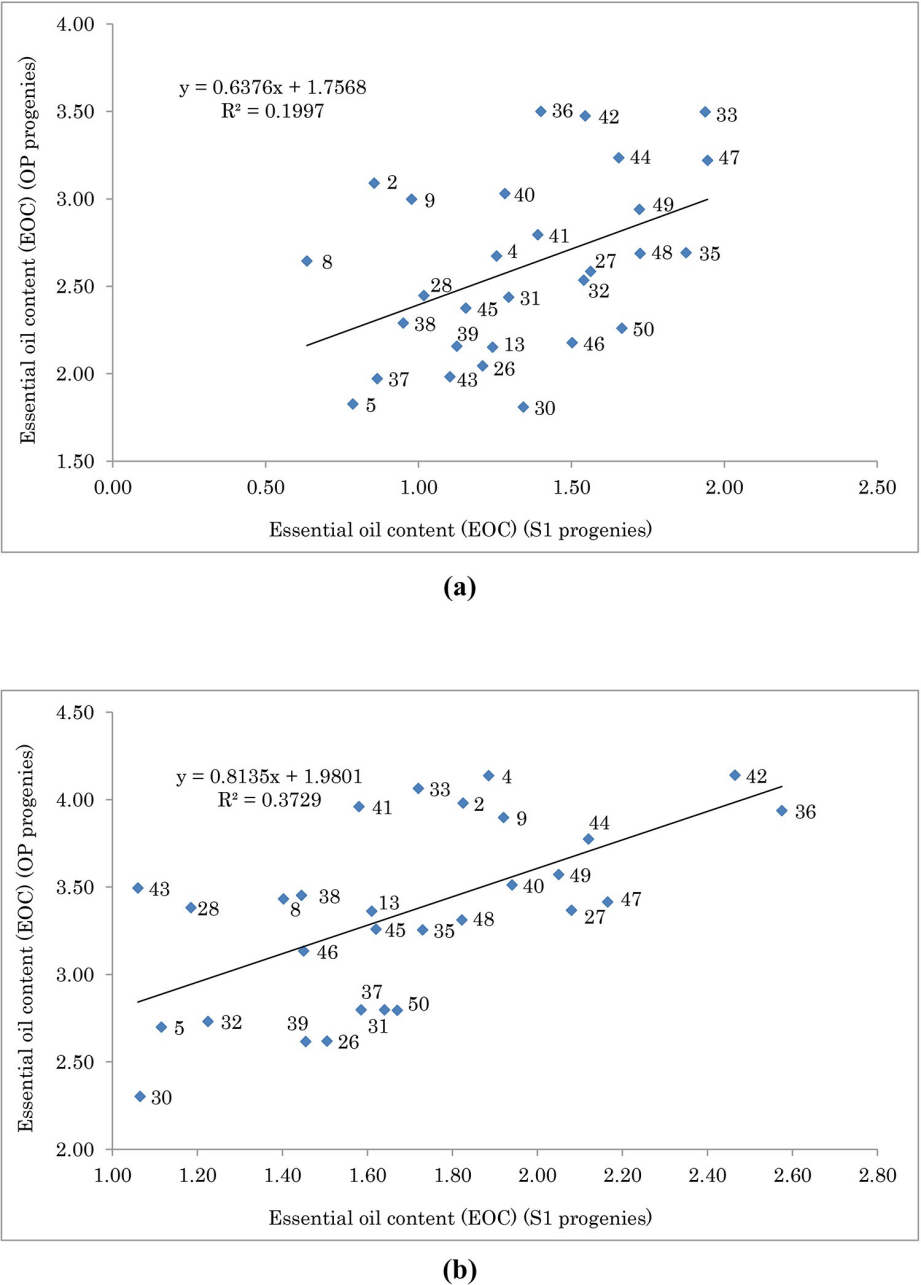
Essential oil content (EOC) of 29 selfed (S1) and open-pollinated (OP) populations of fennel under normal (a) and water deficit (b) conditions. The F-test indicated that the model is not significant for normal condition, and for water deficit condition is significant in 0.05 probability level.

**Fig 3:**
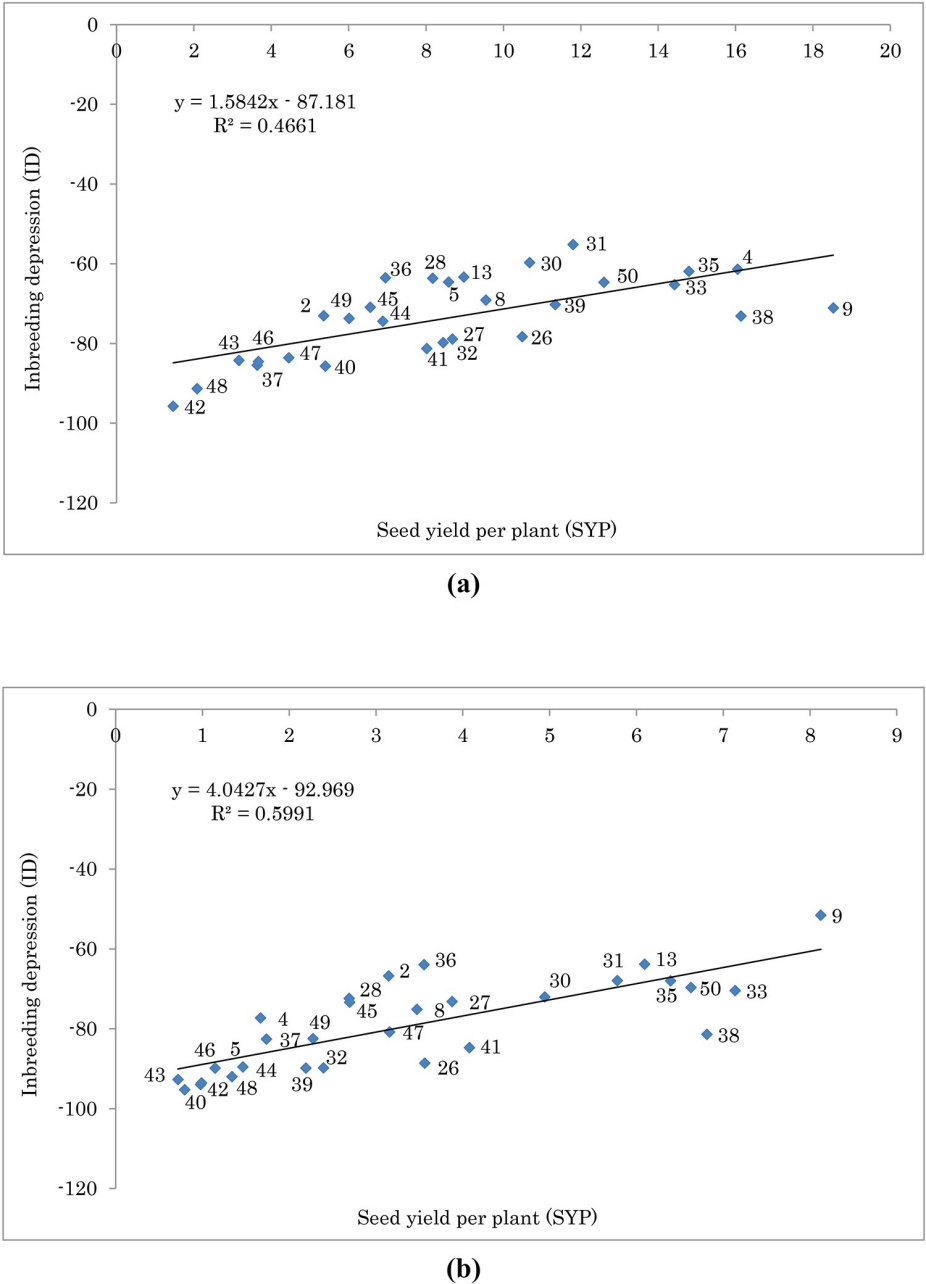
Biplot of seed yield per plant (SYP) vs. inbreeding depression (ID) for S1 population of fennel under (a) normal condition and (b) water deficit condition. There was significant association between SYP and ID under normal (r = 0.68 **) and water deficit (r = 0.77 **) conditions. The F-test indicated that the model is significant for normal and water deficit conditions in 0.01 probability level.

**Fig 4:**
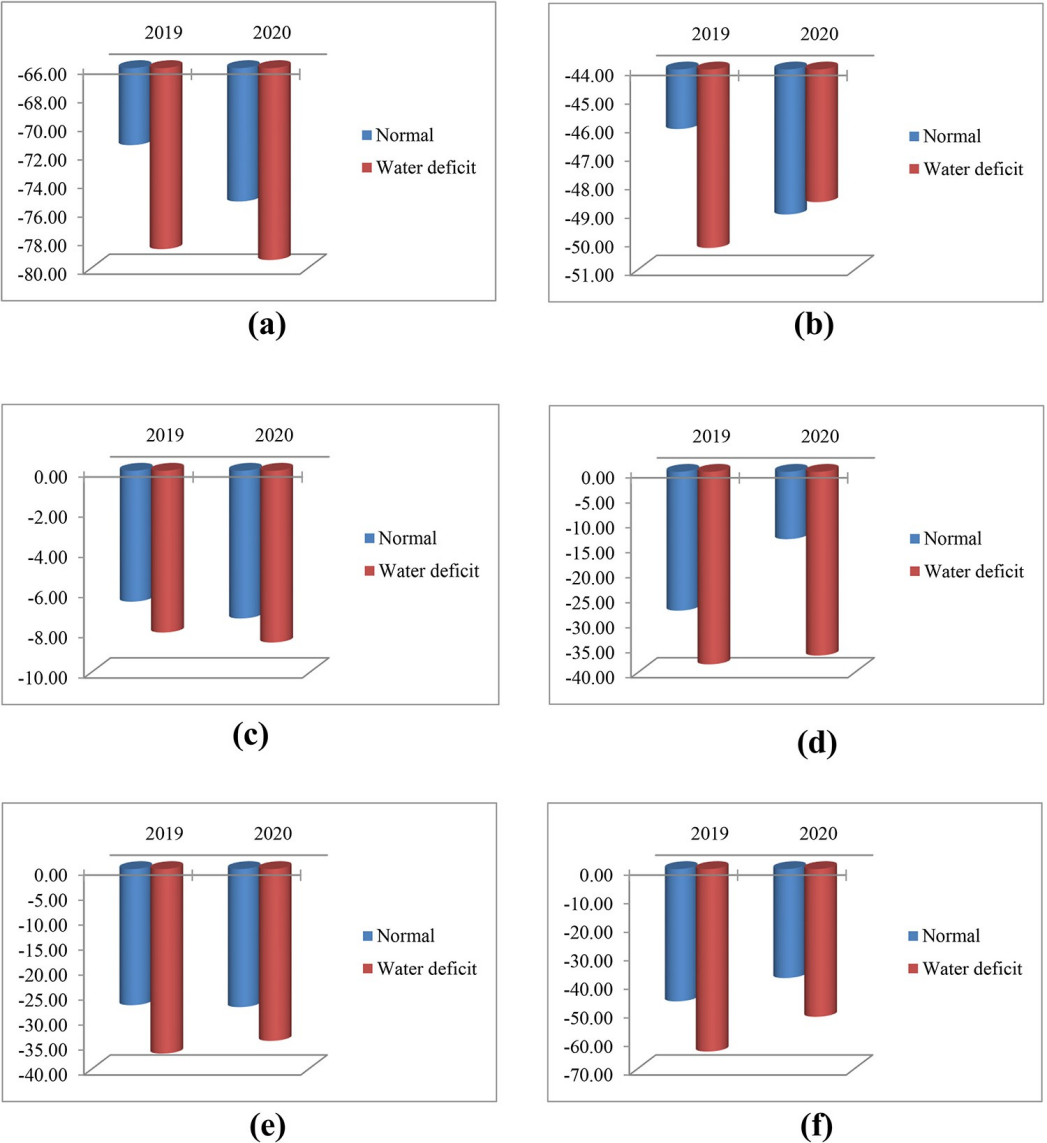
Percentage of inbreeding depression for (a) seed yield per plant (SYP); (b) essential oil content (EOC); (c) relative water content (RWC); (d) carotenoid content (CAR); (e) chlorophyll *a* content (Chl *a*); and (f) chlorophyll *b* content (Chl *b*) under two years (2019 and 2020) and two moisture conditions (normal and water deficit).

Figs 1 and 2 show the relationship between SYP and EOC of S1 and OP progenies, respectively. Genotypes with higher means for S1 showed lower inbreeding depression. Fig 3 shows the relationship between ID and SYP of S1 progenies. Genotypes with higher S1 yield and lower ID can be selected as better ones. The interaction effect of year and moisture conditions on ID for some important traits is given in Fig 4. As shown, for all traits and in both years the values of ID under normal condition were higher than water deficit, except for the year 2019 in EOC (Fig 4B). Moreover, under normal and water deficit conditions, the highest value of ID was observed for SYP (Fig 4A) and the lowest value was detected for RWC (Fig 4C).

The effect of water deficit on all the measured traits in each population is given in Table 2. In both populations, the magnitude of mean performance was decreased for most of the measured traits under water-deficit condition. However, some of the traits including EOC, HI, and PRO were increased in both populations under water deficit. The ratio of TChl/CAR significantly increased due to water deficit in S1 population, but it was not affected in OP one. In OP population, DG significantly increased under water deficit; while it was not affected in S1 one (Table 2). The magnitude of mean performance of Chl *a*/Chl *b* was not affected under water deficit condition. As expected, SYP drastically reduced by water deficit in both populations, so that this reduction was on average 61 and 57% in S1 and OP populations, respectively. Similarly, under water deficit, EOC was decreased compared with normal condition. These reductions were approximately 28 and 35%, in S1 and OP populations, respectively (Table 2).

**Fig 5:**
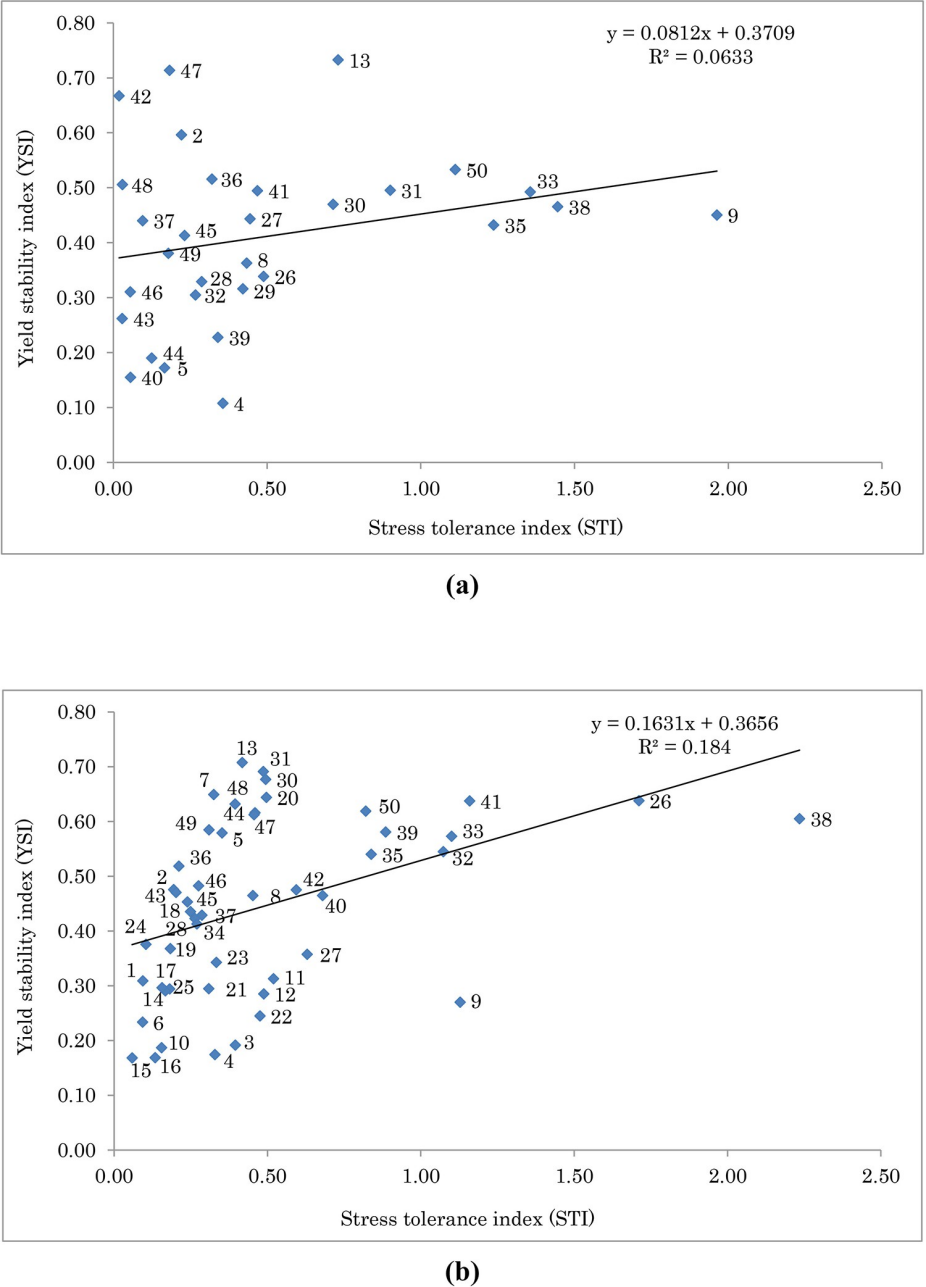
Biplot of stress tolerance index (STI) vs. yield stability index (YSI) for (a) S1 and (b) OP populations of fennel. The F-test indicated that the model is not significant for normal and water deficit conditions.

**Fig 6:**
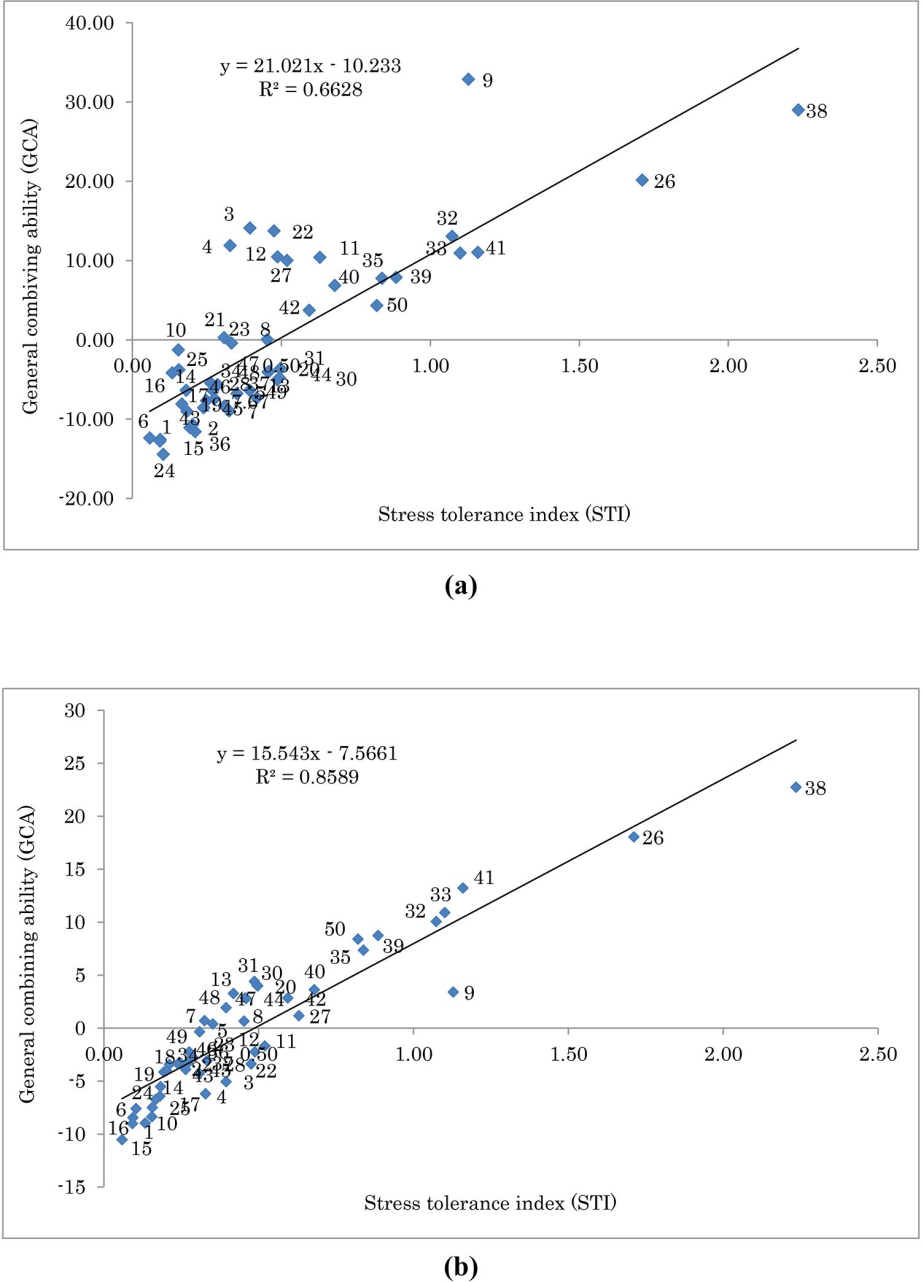
Biplot of general combining ability (GCA) vs. stress tolerance index (STI) for 49 open-pollinated (OP) populations of fennel under normal (a) and water deficit (b) conditions. The F-test indicated that the model is significant for normal and water deficit conditions in 0.01 probability level.

To assess drought tolerance of the fennel populations, three selection indices of STI, TOL, and YSI were calculated. The relationship between STI and YSI of S1 and OP populations and distribution of selfed and open-pollinated progenies is presented in Fig 5. As a result, in S1 population the higher values of STI were obtained for families 9, 38, 33, 35, 50, and 31, and the lower values were detected for families 42, 43, 48, 40, 46, and 37, respectively. The higher values for YSI were obtained for families 13, 47, 42, 2, and 50, and the lower values of this index were observed for families 4, 40, 5, 44, and 39, respectively (Fig 5A). In OP population, the higher values of STI were obtained for families 38, 26, 41, 9, 33, and 32, and the lower values were detected for families 15, 6, 1, 24, 16, and 10, respectively. The higher values for YSI were obtained for families 13, 31, 30, 7, and 20, and the lower values of this index were detected for families 15, 16, 4, 10, and 3, respectively (Fig 5B). The results of linear regression analysis (Fig 6) showed a significant and positive relationship between GCA and STI under normal (*R*2 = 0.66, *p* > 0.01; Fig 6A) and water deficit (*R*2 = 0.86, *p* > 0.01; Fig 6B) conditions. As a result, it was possible to identify genotypes having high values of combining ability and drought tolerance. In this respect, genotypes 26 and 38 were identified as the superior ones under both moisture conditions (Fig 6A and 6B).

### Variance components and heritability

The estimates of broad-sense heritability of S1 population, narrow-sense heritability of OP population, and variance components of evaluated traits were calculated for each moisture environments, separately (Table 5). According to the results, in S1 population, heritability estimates ranged from 23.41% (for UU) to 72.94% (for DG) under normal condition and from 34.44% (for UU) to 71.26% (for EOC) under water deficit condition. In the OP population, these ranges were from 23.22% (for SU) to 70.10% (for DF) under normal condition and from 24.32% (for SU) to 71.88% (for EOC) under water deficit condition (Table 5).

**Table 5:** Estimates of variance components, broad-sense heritability (*h2b*) of S1 population, and narrow-sense heritability (*h2n)* of OP population for the evaluated traits of fennel under two moisture conditions (normal and water deficit) during 2019–2020.

	Selfed genotypes (S1)								Open-pollinated genotypes (OP)							
	Normal environment				Water stress environment				Normal environment				Water stress environment			
Traits	*σ* ^*2*^_*g*_	*σ* ^*2*^_*gy*_	*σ* ^*2*^_*e*_	*h* ^*2*^_*b*_	*σ* ^*2*^_*g*_	*σ* ^*2*^_*gy*_	*σ* ^*2*^_*e*_	*h* ^*2*^_*b*_	*σ* ^*2*^_*g*_	*σ* ^*2*^_*gy*_	*σ* ^*2*^_*e*_	*h* ^*2*^_*n*_	*σ* ^*2*^_*g*_	*σ* ^*2*^_*gy*_	*σ* ^*2*^_*e*_	*h* ^*2*^_*n*_
DG	0.33	0.14	0.22	72.94	0.52	0.14	0.33	71.05	1.19	0.68	1.75	60.54	1.95	1.41	0.67	60.16
DF	6.08	3.72	3.14	69.72	9.18	6.31	1.42	67.12	6.35	2.03	6.78	70.10	6.91	3.28	2.83	69.10
DM	13.00	29.76	1.78	45.08	21.36	36.10	7.34	51.79	7.70	6.09	2.57	64.55	10.62	7.81	4.53	67.85
PHT	17.30	9.78	7.12	69.38	23.85	8.02	10.53	60.71	60.46	49.01	29.03	62.39	37.49	42.61	29.83	55.87
FW	575.71	404.32	48.95	69.88	194.42	140.43	67.45	67.28	33987.05	48999.78	10194.68	55.66	2199.11	2889.34	318.65	57.98
DW	71.82	82.49	2.44	63.18	8.15	8.06	2.08	62.82	1719.74	2924.67	497.93	52.01	137.24	242.40	11.15	52.06
UP	3.84	3.37	0.61	65.81	1.86	1.11	0.99	69.47	9.70	14.93	13.54	45.95	6.96	5.60	3.56	60.30
UU	0.48	1.57	3.20	23.41	0.75	0.57	2.29	34.44	0.98	3.32	3.18	23.56	0.54	0.25	4.06	32.33
SU	3.52	3.46	8.84	47.18	1.43	0.72	5.01	47.00	3.38	17.19	7.38	23.22	1.90	4.37	12.38	24.32
SYP	1.76	3.21	0.37	44.18	0.22	0.31	0.21	47.32	15.00	35.60	13.23	40.94	3.50	5.02	2.87	45.91
HI	19.78	14.20	2.98	67.43	47.11	36.96	25.85	63.57	55.68	52.51	25.22	62.90	55.06	99.41	17.89	48.39
TSW	0.018	0.008	0.004	70.19	0.021	0.003	0.017	67.78	0.076	0.046	0.026	62.01	0.079	0.078	0.016	62.06
EOC	0.025	0.022	0.002	67.73	0.020	0.008	0.013	71.26	0.058	0.022	0.049	69.49	0.048	0.010	0.039	71.88
SL	0.04	0.02	0.01	68.97	0.06	0.05	0.01	64.94	0.10	0.06	0.09	59.57	0.09	0.08	0.02	62.14
SW	0.002	0.002	0.001	66.73	0.003	0.001	0.001	66.87	0.007	0.007	0.006	53.03	0.007	0.007	0.003	57.65
RWC	2.64	3.27	2.53	51.04	3.39	2.00	1.03	66.40	4.29	-0.01^†^	9.47	64.46	4.17	2.46	2.06	69.13
PRO	0.00003	0.00001	0.00002	70.25	0.00018	0.00014	0.00017	61.67	0.00002	0.00002	0.00003	53.23	0.00003	0.00003	0.00006	40.71
Chl *a*	0.083	0.030	0.054	69.61	0.033	0.002	0.027	70.57	0.129	-0.106^†^	0.329	61.08	0.080	0.009	0.023	65.54
Chl *b*	0.0124	0.0087	0.0084	55.28	0.0015	-0.0001	0.0028	68.20	0.0128	0.0097	0.0151	34.33	0.0028	0.0001	0.0021	67.83
CAR	0.015	0.004	0.010	65.71	0.013	0.004	0.006	65.46	0.016	-0.014^†^	0.053	54.51	0.013	0.002	0.008	58.26
TChl	0.13	0.05	0.06	68.50	0.05	0.01	0.02	69.71	0.16	-0.09^†^	0.33	65.25	0.09	0.01	0.03	65.69
Chl *a*/Chl *b*	4.22	12.51	7.50	32.09	73.25	94.82	25.57	56.97	1.44	0.31	6.92	42.86	5.07	0.48	2.53	68.43
TChl/CAR	0.12	0.09	0.11	54.41	333.05	-24.85^†^	409.75	48.13	0.07	-0.11^†^	0.42	39.08	0.13	0.07	0.14	48.56

*σ*^*2*^_*g*_ is the genotype, *σ*^*2*^_*gy*_ is the genotype × year, and *σ*^*2*^_*e*_ is the error variance. *h*^*2*^_*b*_ is the broad-sense heritability, *h*^*2*^_*n*_ is the narrow-sense heritability.

† Values assumed to be zero for estimating of heritability.

CAR, Carotenoid content; Chl *a*, Chlorophyll *a* content; Chl *b*, Chlorophyll *b* content; Chl *a*/Chl *b*, Ratio of chlorophyll *a* to chlorophyll *b* content; DF, Days to flowering; DG, Days to germination; DM, Days to maturity; DW, Plant dry weight; EOC, Essential oil content; FW, Plant fresh weight; HI, Harvest index; PHT, Plant height; PRO, Proline content; RWC, Relative water content; SL, Seed length; SU, Number of seeds per umbelets; SW, Seed width; SYP, Seed yield per plant; TChl, Total chlorophyll content; TChl/CAR, Ratio of total chlorophyll to carotenoid content; TSW, Thousand seed weight; UP, Number of umbels per plant; UU, Number of umbelets per umbel.

Results showed that for all traits except for DF, DM, RWC, and Chl *a*/Chl *b*, estimates of heritability were higher in S1 population than in OP one. Moreover, in S1 population some of the studied traits had higher heritability estimates under normal condition and some of them showed higher heritability under water deficit. In OP population, except for DG, DF, PHT, HI, and RWC heritability estimates of all traits were higher under water deficit condition than the normal one. Results also revealed that, under both moisture conditions, the heritability estimates of yield components were higher than that of seed yield (Table 5).

### Association among traits and biplot analysis

Phenotypic correlation coefficients, estimated on the means of data from two years in normal and water deficit conditions for S1 and OP populations, are given in Tables 6 and 7. Results showed that, in S1 population, UP and HI were positively correlated with SYP, under normal and water deficit conditions (Table 6). In OP population, SYP had significant and positive associations with FW, DW, UP, UU, and SU under normal condition; while under water deficit it was positively associated with DG, DF, PHT, DW, UP, UU, SU, TSW, SL, and SW (Table 7). In OP population, significant and positive correlations were observed between DG, DF, CAR, and EOC under normal and water deficit conditions; while this was not the case in S1 population (Tables 6 and 7). In S1 population, there was a significant and positive correlation between CAR and EOC under water deficit condition; meanwhile, in OP population this association was observed under normal condition. According to the results, under normal and water deficit conditions phenological traits of DG and DF had significant and positive correlations with FW and UP in S1 population; while in OP one, these traits were positively associated with UP, UU, SU, TSW, and SW. In both groups of progenies (S1 and OP) and under both moisture conditions, there were significant and positive associations between photosynthetic pigments including Chl *a*, Chl *b*, TChl, CAR, and TChl/CAR. However, these traits showed no significant associations with SYP and its components (Tables 6 and 7).

**Table 6:** Correlation coefficients among morphological, agronomic, and physiological traits of selfed (S1) population of fennel in normal (above diagonal) and water deficit condition (below diagonal).

	DG	DF	DM	PHT	FW	DW	UP	UU	SU	SYP	HI	TSW
DG	1	0.82**	0.08	0.46*	0.41*	0.10	0.52**	0.25	0.39*	0.19	0.18	0.12
DF	0.87**	1	0.25	0.30	0.47**	0.19	0.42*	0.34	0.34	0.08	0.02	0.14
DM	0.31	0.34	1	0.01	-0.05	-0.09	0.20	-0.02	0.03	0.18	0.28	-0.01
PHT	0.48**	0.41*	0.11	1	0.23	0.11	0.25	0.14	0.04	0.31	0.16	-0.34
FW	0.24	0.35	0.42*	0.13	1	0.32	0.50**	-0.02	0.02	0.35	0.14	0.27
DW	-0.18	-0.15	0.28	0.27	0.44*	1	-0.13	-0.13	-0.06	0.21	-0.56**	-0.09
UP	0.58**	0.46*	0.25	0.27	0.04	-0.22	1	0.03	0.24	0.47**	0.51**	0.36
UU	0.20	0.28	-0.16	0.06	-0.38*	-0.35	0.23	1	0.11	-0.20	-0.01	-0.12
SU	0.25	0.21	0.12	-0.07	-0.01	0.03	0.35	0.33	1	0.21	0.15	0.08
SYP	0.25	0.19	0.18	0.25	0.37*	0.33	0.45*	-0.18	0.02	1	0.64**	0.15
HI	0.48**	0.41*	-0.02	0.05	0.06	-0.47**	0.67**	0.14	0.07	0.61**	1	0.20
TSW	-0.07	-0.24	-0.03	-0.36	-0.11	-0.04	0.02	-0.24	-0.19	-0.12	0.03	1
EOC	0.08	0.01	0.26	0.03	0.09	0.13	-0.01	-0.23	0.03	-0.03	-0.10	0.02
SL	-0.12	0.04	0.04	-0.13	-0.06	0.16	-0.09	0.12	0.11	-0.01	-0.08	-0.07
SW	-0.16	-0.17	-0.31	-0.13	-0.03	-0.01	-0.08	0.04	-0.15	-0.03	-0.07	0.21
RWC	-0.08	-0.07	0.17	-0.03	0.03	0.00	-0.05	-0.18	-0.23	0.09	0.09	0.12
PRO	-0.08	-0.28	-0.54**	0.07	-0.05	0.01	0.13	-0.10	0.03	0.17	0.07	-0.04
Chl *a*	0.41*	0.42*	0.19	-0.07	0.22	-0.21	0.16	0.06	0.33	-0.09	0.18	-0.06
Chl *b*	0.25	0.27	0.13	-0.14	0.08	-0.22	-0.06	-0.07	-0.02	0.05	0.20	-0.05
CAR	0.35	0.35	0.23	0.13	0.22	-0.12	0.11	-0.03	-0.01	-0.01	0.18	-0.04
TChl	0.42*	0.43*	0.19	-0.09	0.21	-0.22	0.14	0.04	0.30	-0.07	0.20	-0.06
Chl *a*/Chl *b*	-0.03	-0.06	-0.13	0.15	0.08	0.27	0.21	0.08	0.26	0.09	-0.08	-0.07
TChl/CAR	-0.01	-0.04	-0.17	0.18	0.06	0.26	0.07	0.01	0.17	0.34	0.02	-0.31
	EOC	SL	SW	RWC	PRO	Chl *a*	Chl *b*	CAR	TChl	Chl *a*/chl *b*	TChl/CAR	
DG	-0.28	0.06	0.08	0.09	-0.11	0.24	0.29	0.16	0.28	-0.15	0.11	
DF	-0.11	0.30	0.18	0.22	-0.15	0.18	0.33	0.21	0.24	-0.13	0.01	
DM	0.07	0.17	0.05	0.19	-0.17	0.23	-0.13	0.32	0.17	0.37*	-0.10	
PHT	-0.26	-0.34	-0.39*	0.29	0.21	0.21	0.01	0.20	0.18	0.11	-0.03	
FW	-0.37*	0.34	0.19	0.20	0.22	-0.31	0.09	-0.30	-0.25	-0.17	-0.02	
DW	0.24	0.20	0.05	0.13	0.08	-0.15	0.06	-0.12	-0.12	0.06	-0.04	
UP	-0.37*	0.32	0.11	-0.09	0.01	-0.13	0.13	-0.22	-0.08	-0.06	0.09	
UU	-0.27	0.01	-0.02	0.10	0.08	0.04	0.18	0.08	0.08	-0.25	-0.12	
SU	-0.01	0.43*	0.24	0.02	-0.17	0.19	0.39*	0.04	0.26	-0.31	0.24	
SYP	-0.08	0.28	0.08	0.11	0.12	-0.22	-0.07	-0.34	-0.21	0.04	0.08	
HI	-0.25	0.06	0.02	0.04	-0.01	-0.09	-0.15	-0.22	-0.12	0.05	0.08	
TSW	0.04	0.44*	0.53**	-0.06	-0.14	-0.20	0.16	-0.23	-0.14	-0.18	0.03	
EOC	1	-0.08	-0.13	0.03	-0.27	0.11	-0.10	0.23	0.07	0.18	-0.16	
SL	-0.11	1	0.53**	-0.13	-0.27	-0.35	0.19	-0.37*	-0.26	-0.27	0.02	
SW	-0.44*	0.43*	1	-0.04	0.07	0.06	0.50**	-0.09	0.17	-0.31	0.33	
RWC	0.26	0.27	0.33	1	-0.10	0.36	0.16	0.32	0.35	0.03	0.17	
PRO	-0.27	-0.19	0.38*	-0.17	1	-0.24	0.02	-0.15	-0.21	-0.12	-0.20	
Chl *a*	0.41*	0.06	-0.07	0.28	-0.21	1	0.47**	0.86**	0.98**	0.13	0.46*	
Chl *b*	0.19	0.10	0.01	0.35	-0.31	0.47**	1	0.17	0.65**	-0.69**	0.73**	
CAR	0.39*	-0.10	-0.39*	0.02	-0.22	0.63**	0.20	1	0.78**	0.34	-0.03	
TChl	0.41*	0.07	-0.06	0.31	-0.24	0.99**	0.59**	0.61**	1	-0.06	0.57**	
Chl *a*/Chl *b*	0.08	0.08	0.21	-0.02	0.40*	0.17	-0.64**	-0.04	0.05	1	-0.44*	
TChl/CAR	0.04	0.09	0.25	0.14	0.34	-0.06	-0.15	-0.55**	-0.08	0.52**	1	

* and ** show significance at the 0.05 and 0.01 probability levels, respectively.

CAR, Carotenoid content; Chl *a*, Chlorophyll *a* content; Chl *b*, Chlorophyll *b* content; Chl *a*/Chl *b*, Ratio of chlorophyll *a* to chlorophyll *b* content; DF, Days to flowering; DG, Days to germination; DM, Days to maturity; DW, Plant dry weight; EOC, Essential oil content; FW, Plant fresh weight; HI, Harvest index; PHT, Plant height; PRO, Proline content; RWC, Relative water content; SL, Seed length; SU, Number of seeds per umbelets; SW, Seed width; SYP, Seed yield per plant; TChl, Total chlorophyll content; TChl/CAR, Ratio of total chlorophyll to carotenoid content; TSW, Thousand seed weight; UP, Number of umbels per plant; UU, Number of umbelets per umbel.

**Table 7:** Correlation coefficients among morphological, agronomic, and physiological traits of open-pollinated (OP) population of fennel in normal (above diagonal) and water deficit condition (below diagonal).

**Traits**	DG	DF	DM	PHT	FW	DW	UP	UU	SU	SYP	HI	TSW
DG	1	0.88**	-0.05	0.07	0.06	0.03	0.30*	0.17	0.43**	0.08	-0.07	0.35*
DF	0.90**	1	-0.03	0.12	0.15	0.15	0.44**	0.34*	0.53**	0.19	-0.17	0.36*
DM	0.03	0.08	1	0.01	0.03	0.25	0.01	-0.01	-0.08	-0.26	-0.35*	0.01
PHT	0.32*	0.38**	0.13	1	0.26	0.03	0.22	0.15	0.01	0.28	0.05	-0.30*
FW	0.14	0.23	0.34*	0.04	1	0.50**	0.38**	0.34*	0.32*	0.34*	-0.46**	-0.01
DW	0.26	0.25	0.12	0.23	0.44**	1	0.24	0.34*	0.26	0.29*	-0.83**	0.13
UP	0.31*	0.45**	0.08	0.28	0.23	0.38**	1	0.38**	0.35*	0.67**	0.01	0.24
UU	0.19	0.19	0.05	0.11	0.04	0.16	0.04	1	0.57**	0.44**	-0.23	0.03
SU	0.10	0.07	-0.09	0.15	-0.17	-0.19	-0.11	0.06	1	0.34*	-0.21	0.09
SYP	0.51**	0.43**	0.01	0.30*	0.18	0.62**	0.41**	0.29*	0.00	1	0.07	0.04
HI	0.08	0.02	-0.09	0.02	-0.50**	-0.64**	-0.16	0.05	0.16	0.06	1	-0.06
TSW	0.42**	0.46**	-0.19	-0.03	-0.06	0.28	0.55**	0.09	-0.05	0.46**	0.01	1
EOC	0.35*	0.40**	-0.02	0.39**	-0.15	0.03	0.26	0.21	-0.08	0.12	0.16	0.16
SL	0.15	0.18	0.04	-0.01	-0.05	0.31*	0.25	0.02	-0.12	0.31*	-0.25	0.57**
SW	0.29*	0.33*	0.06	0.13	0.12	0.51**	0.51**	0.06	-0.19	0.52**	-0.25	0.66**
RWC	0.11	0.20	0.11	0.19	-0.03	0.14	0.24	-0.04	-0.30*	0.18	0.07	0.06
PRO	-0.19	-0.16	-0.08	-0.01	0.19	0.14	-0.19	0.19	0.33*	0.07	-0.14	-0.28*
Chl *a*	0.16	0.12	0.05	0.39**	-0.02	0.23	0.25	0.15	0.01	0.21	-0.07	-0.01
Chl *b*	0.06	0.01	-0.26	-0.02	-0.08	-0.07	-0.24	0.14	0.17	0.01	0.01	-0.02
CAR	-0.05	-0.10	0.05	0.05	-0.03	0.18	-0.04	0.15	0.01	0.03	-0.17	-0.27
TChl	0.16	0.11	0.01	0.37**	-0.03	0.21	0.19	0.17	0.04	0.20	-0.06	-0.02
Chl *a*/Chl *b*	-0.12	-0.10	0.08	0.24	0.02	0.32*	0.24	-0.02	-0.19	0.15	-0.12	-0.12
TChl/CAR	0.22	0.20	-0.08	0.40**	-0.09	0.05	0.26	0.10	0.10	0.23	0.20	0.29*
**Traits**	EOC	SL	SW	RWC	PRO	Chl *a*	Chl *b*	CAR	TChl	Chl *a*/Chl *b*	TChl/CAR	
DG	0.41**	-0.02	-0.21	-0.12	0.02	-0.23	0.02	0.03	-0.18	-0.13	-0.30*	
DF	0.49**	0.05	-0.15	-0.01	0.06	-0.14	0.09	0.14	-0.09	-0.18	-0.31*	
DM	0.05	0.03	-0.03	0.10	0.04	0.01	0.02	0.10	0.01	-0.15	-0.13	
PHT	0.27	-0.14	-0.06	0.24	-0.21	0.12	0.02	0.10	0.11	-0.07	-0.01	
FW	-0.01	0.09	-0.22	-0.07	-0.18	-0.15	-0.02	0.06	-0.13	-0.10	-0.24	
DW	0.06	0.17	-0.03	-0.03	0.01	0.01	0.04	0.16	0.02	0.05	-0.15	
UP	0.26	-0.06	0.02	-0.08	0.05	0.03	0.18	0.14	0.07	-0.03	-0.08	
UU	0.36*	0.21	-0.01	0.13	-0.07	0.09	0.32*	0.21	0.16	-0.29*	-0.03	
SU	0.24	0.11	-0.02	-0.12	0.06	0.02	0.27	0.11	0.09	-0.25	0.01	
SYP	0.25	-0.01	-0.06	-0.10	0.04	0.10	0.23	0.11	0.15	-0.03	0.06	
HI	-0.07	-0.19	0.17	-0.04	0.01	-0.01	0.07	-0.23	0.01	-0.08	0.29*	
TSW	0.08	0.48**	0.26	-0.35*	0.11	-0.06	0.03	0.17	-0.04	0.04	-0.25	
EOC	1	0.08	-0.16	0.24	-0.02	0.09	0.21	0.29*	0.13	-0.15	-0.21	
SL	0.00	1	0.43**	-0.02	-0.02	0.04	-0.02	0.16	0.03	0.05	-0.19	
SW	0.03	0.77**	1	0.05	0.16	0.01	0.12	0.13	0.04	-0.04	-0.06	
RWC	0.33*	0.13	0.12	1	-0.23	0.11	0.04	0.21	0.10	-0.22	-0.15	
PRO	-0.09	-0.30*	-0.18	-0.19	1	0.07	0.23	0.12	0.12	0.01	0.02	
Chl *a*	0.22	0.14	0.30*	0.14	-0.18	1	0.54**	0.74**	0.97**	0.14	0.51**	
Chl *b*	0.04	-0.05	0.01	0.05	0.05	0.13	1	0.40**	0.71**	-0.61**	0.53**	
CAR	0.17	0.03	0.01	0.11	0.08	0.72**	0.11	1	0.72**	0.14	-0.15	
TChl	0.21	0.12	0.28*	0.15	-0.16	0.98**	0.32*	0.71**	1	-0.05	0.56**	
Chl *a*/Chl *b*	0.07	0.09	0.09	0.18	-0.01	0.55**	-0.57**	0.54**	0.42**	1	-0.20	
TChl/CAR	0.09	0.07	0.31*	0.04	-0.29*	0.43**	0.29*	-0.26	0.47**	-0.05	1	

* and ** show significance at the 0.05 and 0.01 probability levels, respectively.

CAR, Carotenoid content; Chl *a*, Chlorophyll *a* content; Chl *b*, Chlorophyll *b* content; Chl *a*/Chl *b*, Ratio of chlorophyll *a* to chlorophyll *b* content; DF, Days to flowering; DG, Days to germination; DM, Days to maturity; DW, Plant dry weight; EOC, Essential oil content; FW, Plant fresh weight; HI, Harvest index; PHT, Plant height; PRO, Proline content; RWC, Relative water content; SL, Seed length; SU, Number of seeds per umbelets; SW, Seed width; SYP, Seed yield per plant; TChl, Total chlorophyll content; TChl/CAR, Ratio of total chlorophyll to carotenoid content; TSW, Thousand seed weight; UP, Number of umbels per plant; UU, Number of umbelets per umbel.

**Fig 7:**
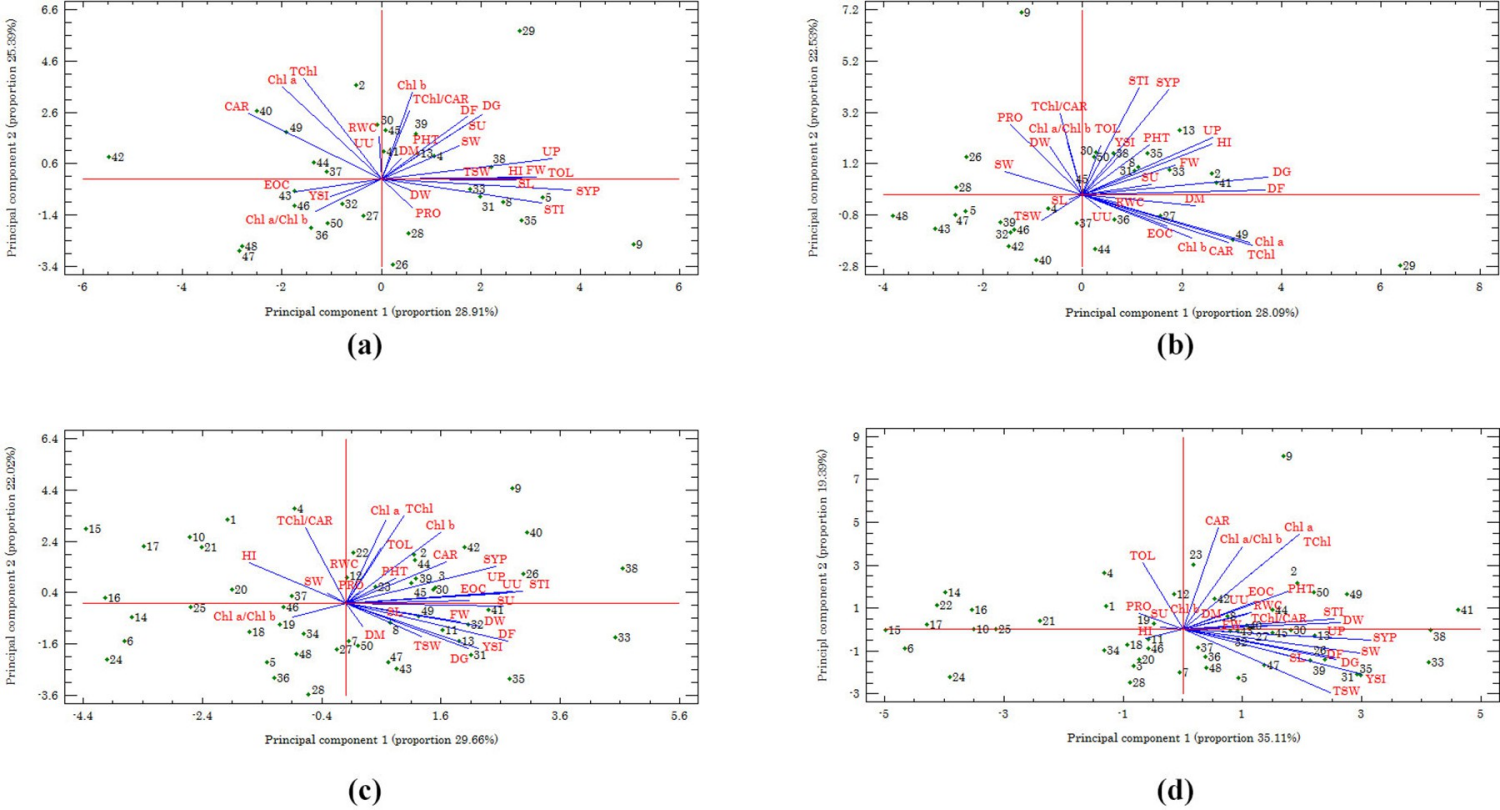
Distribution of the first two principal components (PC) of phenological, morphological, physiological traits, and selection indices of fennel under normal and deficit irrigations in 30 selfed (S1) (Fig 7A and 7B) and 49 open-pollinated populations (Fig 7C and 7D). CAR, Carotenoid content; Chl *a*, Chlorophyll *a* content; Chl *b*, Chlorophyll *b* content; Chl *a*/Chl *b*, Ratio of chlorophyll *a* to chlorophyll *b* content; DF, Days to flowering; DG, Days to germination; DM, Days to maturity; DW, Plant dry weight; EOC, Essential oil content; FW, Plant fresh weight; HI, Harvest index; PHT, Plant height; PRO, Proline content; RWC, Relative water content; SL, Seed length; STI, Stress tolerance index; SU, Number of seeds per umbelets; SW, Seed width; SYP, Seed yield per plant; TChl, Total chlorophyll content; TChl/CAR, Ratio of total chlorophyll to carotenoid content; TOL, Tolerance index; TSW, Thousand seed weight; UP, Number of umbels per plant; UU, Number of umbelets per umbel; YSI, Yield stability index.

A multivariate technique (i.e. PCA) was used to reduce the dimension of data and view the relationship between traits and genotypes. In S1 population, the first two components justified approximately 54% and 51% of the total variation under the normal and water deficit conditions, respectively (Fig 7A and 7B). Under normal condition, the first principal component (PC1) was positively correlated with FW, UP, SYP, HI, SL, TOL, and STI, and negatively associated with CAR. Therefore, this component was considered as the ‘‘productivity and drought tolerance component”. The second principal component (PC2) had positive correlations with DG, DF, Chl *a*, Chl *b*, CAR, TChl, and TChl/CAR. As higher values of these traits can lead to a long maturity period, and higher photosynthetic capacity, hence PC2 was called the ‘‘maturity and photosynthetic pigments component”. Thus, improvements in productivity, photosynthetic capacity, and drought tolerance of fennel can be achieved by selecting genotypes with high values of PC1 and PC2. In this respect, genotypes 9 and 29 were the superior ones. In contrast, genotypes 4, 13, 27, 32, and 41 had low productivity and photosynthetic capacity and were identified as drought sensitive ones (Fig 7A). Under water deficit (Fig 7B), PC1 had positive correlations with DG, DF, DM, UP, HI, Chl *a*, Chl *b*, CAR, and TChl; therefore it was named ‘‘maturity and photosynthetic pigments component”. Moreover, PC2 was positively correlated with SYP, PRO, TChl/CAR, and STI and was considered as “productivity, drought tolerance, and photosynthetic capacity component”. Therefore, selection based on high values of PC1 and PC2 can lead to improvement of productivity, photosynthetic capacity, and drought tolerance of fennel. In this respect, genotypes 13 and 29 were the superior ones (Fig 7B).

In OP population, the first two components explained approximately 52% and 55% of the total variation under normal and water deficit conditions, respectively (Fig 7C and 7D). Under normal condition (Fig 7C), PC1 was positively correlated with DG, DF, FW, DW, UP, UU, SU, SYP, EOC, YSI, and STI, and was called the “productivity, stability and drought tolerance component”. On the other hand, PC2 had positive correlations with Chl *a*, Chl *b*, TChl, TChl/CAR, and TOL. As higher values of these traits lead to higher photosynthetic capacity and drought tolerance, hence PC2 was named ‘photosynthetic pigments component’. In total, improvements in productivity, photosynthetic capacity, yield stability, and drought tolerance can be made by selecting genotypes high in PC1 and PC2. Therefore, genotypes 9, 15, 24, 35, and 40 can be recommended as suitable genotypes. In contrast, genotypes 8, 12, 19, 23, 37, and 46 have low productivity, photosynthetic capacity, and yield stability, and can be considered as sensitive to water deficit (Fig 7C). Under water deficit (Fig 7D), PC1 had positive correlations with DG, DF, DW, UP, SYP, TSW, SL, SW, Chl *a*, TChl, YSI, and STI; therefore it was considered to be ‘‘potential of seed productivity and drought tolerance component”. Moreover, PC2 was positively correlated with Chl *a*, CAR, TChl, Chl *a*/Chl *b*, and TOL, and negatively associated with TSW; hence it was considered to represent “drought tolerance and photosynthetic capacity”. Therefore, improvement of productivity, drought tolerance, and photosynthetic capacity of fennel may be achieved by the selection of genotypes with high PC1 and PC2. In this respect, genotypes 24, 31, and 35 were the preferable ones.

## Discussion

It is predicted that global warming will affect air temperature, rainfall quantity and distribution, and can result in recurrent droughts in the future [39]. Plants usually respond to water stress with various morphological and physiological changes. Physiological changes have been considered as important adaptation mechanisms for plants to resist water deficit [40]. Moreover, devising and implementing breeding programs require information as to the size and nature of the genetic variation for the various characteristics and their genetic relationship [41]. In the present study, the main focus was on the genetic analysis of performance, drought tolerance, and physiological traits in two different fennel populations with two different pollination systems (S1 and OP). High genotypic variation was found among and within S1 and OP populations for most of the measured traits, emphasizing the high potential for genetic study of these traits. This can facilitate the possibility of selecting genotypes with variable values of specific traits in this germplasm and is in agreement with other studies on fennel [42–44]. Higher estimates of GCV for most of the evaluated traits under water deficit compared with normal condition indicates that water deficit may have increased genetic variation for these traits and selection may be more effective under water deficit. This finding is in contrast to a number of reports stating that genetic progress through selection is usually higher under normal than under water deficit condition [14,45]. However, some researchers have also reported that GCV and the rate of genetic advance through selection were higher under deficit irrigation [46,47]. The reason of this contradiction is not identified; but it may be due to the difference in the environmental condition of experimental site (i.e. location, year and etc.), plant species, genotypes, stress intensity, and duration of stress.

Study of traits related to drought-adaptation and drought-tolerance mechanisms and their responses under stress are important objectives in plant breeding programs. In the current study, water deficit caused a significant reduction in most of the evaluated traits, in both populations. These negative effects could be ascribed to the stomatal closure in response to the low water potential of the soil, decreased rate of photosynthesis, disturbed assimilate partitioning, and disturbance in the grain filling period [8,10]. The region of study (Najafabad, Isfahan, Iran) is a warm and dry area where summer temperature reaches as high as 40°C and precipitation is null; therefore, reductions were expected in most of the evaluated traits. These results agree with the findings of previous studies on other aromatic crops of the Apiaceae family under salt constraint e.g., ajwain (*Trachyspermum ammi* L.) [48], coriander (*Coriandrum sativum* L.) [49], and cumin (*Cuminum cyminum* L.) [9]. Flowering and maturity dates decreased under water deficit, indicating that perennial plants could also escape drought by earlier maturity, similar to annual plants.

The physiological changes have been considered as an important adaptation mechanism for plants to resist drought and could be the result of harmful effects on metabolic processes [40]. In this study, drought stress decreased chlorophyll content. The chlorophyll content is a major determinant of photosynthetic capacity under stress, i.e., higher chlorophyll content and stability of it have been reported to be associated with drought tolerance [50]. Thus, selecting genotypes based on increased or stable chlorophyll content may prevent yield loss under water stress. The decrease in chlorophyll content under water deficit has been discussed as an index of oxidative stress and may be due to pigment photo-oxidation, chlorophyll degradation, reduction of Calvin cycle enzyme activity, and damaged photosynthetic apparatus [51]. Carotenoids can act as non-enzymatic antioxidants and have fundamental roles, such as light harvesting and protection from oxidative damage, caused by drought [52]. Similar to other studies, water deficit led to significant increases in carotenoid and proline content in both populations. Higher carotenoid contents under water stress could be attributed to increased proline and carbohydrates. Decreased or increased carotenoid levels under water stress have been reported in several species [53,54]. Under water deficit, increased proline content in crop species is either due to the prevention of proline oxidation or to the breakdown of proteins [51] and may play a role in maintaining osmotic turgor, protecting and stabilizing membranes and enzymes and thereby preventing electrolyte leakage and bringing concentrations of reactive oxygen species within normal ranges [53,55]. Although there is a strong association between stress intensity and accumulation of proline in higher plants, the relationship between proline accumulation and genetic drought tolerance may not be universal [56].

In breeding for drought tolerance, development of genotypes with high yield under normal condition and less reduction of yield under water deficit is ideal. Selection indices such as STI and TOL can distinguish these tolerant genotypes from others. In this study, several genotypes were identified with higher values of STI from both populations. These genotypes seem to have a higher yield potential under water deficit and may be further improved by crossing to genotypes having higher values of YSI that were stable under both water conditions.

In the present study, except for UU, SU, PRO, Chl *b*, and Chl *a*/Chl *b* under normal condition, and DG, PHT, UU, SU, PRO, CAR, Chl *a*/Chl *b*, and TChl/CAR under water deficit, the S1 population had lower means for all measured traits than the OP population under both moisture conditions, which may be due to the effect of inbreeding depression on these traits. Similar results were reported for fennel by other researchers [27,57]. Inbreeding results in higher degrees of homozygosity which reduces fitness through the increased expression of deleterious recessive alleles or loss of overdominant allele combinations [18]. The results of this study showed that a large variation was observed for inbreeding depression among the genotypes and studied traits. A wide range of inbreeding depression among genotypes for economically important characters was also observed by Nazem et al. [58] in mint. Large genetic variation for inbreeding depression indicates that selection for the low rate of inbreeding is possible in this population [59]. This facilitates the development of the inbred lines for further studies. However, it is necessary to validate the results by further generation.

Estimation of heritability is necessary to design and implement an effective breeding program to maximize genetic improvement; since it provides an indication of the genetic potential available to plant breeders and enables calculation of expected genetic gain for selection in cross-pollinated populations [20,60]. In the S1 progenies, moderate to high values of broad-sense heritability (>0.5) were obtained for all of the studied traits with the exception of UU, SYP, Chl *a*/Chl *b*, and TChl/CAR suggesting the presence of some major genes or QTLs affecting them, and these traits could be improved by recurrent or mass selection [44]. In OP population, the estimates of narrow-sense heritability were moderate to high for all evaluated traits except for UU, SU, SYP, and TChl/CAR, under both conditions, confirms that these traits are mainly under additive genetic control and phenotypic selection can be successful in achieving genetic progress for these traits. These results were generally in agreement with those previously reported in fennel [42,43,61]. In this study, for most of the evaluated traits estimates of heritability in S1 population were higher than in OP one. As the variance among the half-sib progenies represents primarily the additive genetic variance, the heritability which is calculated for OP progenies is an estimation of narrow-sense heritability [21]. However, variance among S1 progenies represents both additive and non-additive genetic variance; therefore, the heritability value represents an estimation of broad-sense heritability, and exactly for this reason the heritability of S1 progenies was higher than the OP ones. Moreover, in S1 progenies estimates of heritability were higher in normal condition than water deficit for some traits, and for the remaining traits its estimates were higher under water deficit condition. While in OP progenies, estimates of *h*2 were higher under water deficit than at normal condition for all traits with the exception of DG, DF, PHT, HI, and RWC, which were advantageous for successful selection in achieving genetic progress and indicate that phenotypic selection under water deficit would be more effective than normal condition. As different genes may contribute to the same trait in different environments therefore, changes in heritability would seem likely to occur with increased or decreased stress [62]. In both populations, low heritability estimates were obtained for the most economically important trait of seed yield under normal and water deficit conditions, which results in lower odds of increasing this trait through direct selection. In these cases, indirect selection through yield components with higher heritability estimates and also high correlation with seed yield would be more effective for achieving the improvement of this trait than direct selection [14]. In the present study, the majority of yield components had higher heritability estimates than seed yield itself. Therefore, determining the relationship between seed yield and its components could lead to effective criteria for indirect selection under normal and water deficit conditions.

Regression of offspring on parents or one parent is a method of estimating heritability that is commonly used by plant breeders. In this study, the heritability estimates based on parent–offspring regression were higher than those based on genetic variance components for most of the traits. The difference between the two methods is due to the inflation of estimations from simple parent–progeny regressions [63]; this is because non-genetic covariances such as genotype × environment interaction include with genetic covariances in the generation of the regression coefficient [64]. Amini et al. [65] in tall fescue and Spanani et al. [59] in orchardgrass calculated heritabilities based on parent–offspring regression, showing that heritabilities in all traits were higher than those based on genetic variance components.

Correlation analysis is a valuable and conclusive analysis for identifying selection criteria for indirectly improving yield potential and economic traits. Highly heritable traits, with easy measurements correlated with complex traits such as essential oil and grain yield, make genotype selection more impressive [66]. Results of phenotypic correlation coefficients and PCA analysis revealed that in S1 population SYP was positively associated with PHT, DW, FW, HI, UP, SU, SW, SL, and TSW under normal condition, and was positively correlated with PHT, FW, HI, UP, and SU under water deficit condition. In OP population, it had positive correlations with PHT, FW, DW, UP, UU, SU, SL, and TSW under normal condition and was positively associated with PHT, FW, DW, UP, UU, SL, SW, and TSW under water deficit. From the positive and significant association of SYP with these traits and moderate to high heritability of them, it appears that these traits could be considered as the main components of seed yield in fennel and indirect selection for them could be effective for improvement of seed yield. Similar to our findings, Singh et al. [67] and Zahid et al. [68] also reported significant and positive correlation between number of umbel and harvest index with grain yield in fennel. Yadav et al. [69] and Kumar et al. [4] reported that seed yield had significant and positive correlations with umbels per plant, the number of umbelets per umbel, and the number of seeds per umbelets in fennel. Contrary to our results regarding the relationships between the yield and its components, Kalleli et al. [70] reported a negative significant correlation between seed yield and 1000-seeds weight in fennel. On the other hand, in both populations STI and YSI showed significant and positive correlations with SYP and its components under normal and water deficit conditions. Therefore, selection for higher plant productivity can result in identifying drought tolerant and stable genotypes under both conditions. Results also revealed that in S1 population, YSI and Chl *a*/Chl *b* under normal condition, and UU, Chl *a*, Chl *b*, TChl, CAR, and RWC under water deficit condition were positively and significantly associated with EOC. In OP population, DM, PHT, FW, DW, UP, UU, SYP, STI, Chl *a*, TChl, Chl *a*/Chl *b*, CAR, TChl/CAR, and RWC under normal condition, and DG, PHT, FW, DW, UP, UU, SU, SL, TSW, STI, YSI, TOL, Chl *a*/Chl *b*, CAR, and TChl under water deficit were positively associated with EOC. Similar results were reported by Lal [71] and Safaei et al. [72] regarding to positive and significant correlation between essential oil content and grain yield of fennel. Shojaiefar et al. [43] reported significant association between essential oil content and plant height which is in agreement with our results. Negative correlations between phenological traits (DG and DF) with seed yield, its components, STI, and YSI in S1 and OP populations suggest that selection for early flowering genotypes can indirectly improve drought tolerance, stability, and seed production of this germplasm under normal and water deficit conditions.

## Conclusions

In conclusion, the substantial genetic variation observed for all evaluated traits between and within the selfed and open-pollinated populations revealed that any changes in plant natural mating systems could clearly change the genetic structure of germplasm. Different levels of inbreeding depression were observed in the present study for the measured traits, which higher values for FW, DW, SYP, EOC, and UP sugges higher heterosis for these traits. Large genetic variations were also observed for inbreeding depression among the progenies, indicating that selection for low inbreeding depression rates is possible in this species while also facilitating the development of inbred lines for future studies. Water deficit could greatly influence agronomic and physiological traits and thus reduced genotypic variation of measured traits. Results of the present study suggest that physiological traits cannot be used as an indicator to distinguish drought-tolerant genotypes in S1 progenies, whereas in OP progenies Chl *a*, Chl *b*, TChl, CAR, PRO, and RWC, which had significant correlations with drought tolerance, may be used for this purpose. Since relatively low heritability was obtained for SYP; both genetic and non-genetic effects play a role in the control of this trait. Therefore, selection based on an index, which is a weighted linear combination of several traits, would be more effective to achieve genetic progress in recurrent selection programs. Moreover, the moderate to high heritability for some of the yield components such as PHT, UP, SW, SL, and TSW suggested that these traits are mainly under additive genetic control, and recurrent selection may be effective to improve the stated traits. These traits were associated with drought tolerance and yield sustainability and could be used in an appropriate selection index to enhance seed yield and identify preferable genotypes for arid and semi-arid regions. Based on the association of STI with GCA, and applying the PCA method, genotypes 9, 13, 29, 38, and 50 from S1 population, and 24, 26, 35, and 38 from OP one were identified as the superior genotypes. They combined higher seed production, yield stability, and drought tolerance, and therefore can be recommended for using in future breeding programs. Further experiments should focus on developing mapping populations for genome studies of agronomic and physiological traits to enhance our knowledge to improve drought tolerance of fennel.

## Supporting information

S1 Table Split-plot in time ANOVA for measured traits in 30 S1 families of fennel evaluated under two moisture conditions (normal and water deficit) during 2 years (2019–2020). (DOC)

S2 Table Split-plot in time ANOVA for measured traits in 49 HS families (OP populations) of fennel evaluated under two moisture conditions (normal and water deficit) during 2 years (2019–2020). (DOC)

S3 Table Mean comparisons of some important agro-morphological characters and essential oil content of S1 population of fennel during 2019–2020 under normal and water deficit conditions. (DOC)

S4 Table Mean comparisons of some important agro-morphological characters and essential oil content of OP population of fennel during 2019–2020 under normal and water deficit conditions. (DOC)

S5 Table General combining ability (GCA) of some important agro-morphological characters and essential oil content of OP population of fennel during 2019–2020 under normal and water deficit conditions. (DOC)

S6 Table Heritability estimates of agro-morphological characters and essential oil content based on parent-offspring regression (h2po) in two populations of fennel. (DOC)

S1 Raw data (XLS)

## Abbreviations

CAR: Carotenoid content
Chl a: Chlorophyll a content
Chl b: Chlorophyll b content
Chl a/Chl b: Ratio of chlorophyll a to chlorophyll b content
DF: Days to flowering
DG: Days to germination
DM: Days to maturity
DW: Plant dry weight
EOC: Essential oil content
FW: Plant fresh weight
GCA: General combining ability
GCV: genetic coefficient of variation
HI: Harvest index
HS: Half-sib
ID: Inbreeding depression
OP: Open-pollinated progenies
PCA: Principal component analysis
PHT: Plant height
PRO: Proline content
RWC: Relative water content
S1: Selfed progenies
SL: Seed length
STI: Stress tolerance index
SU: Number of seeds per umbelets
SW: Seed width
SYP: Seed yield per plant
TChl: Total chlorophyll content
TChl/CAR: Ratio of total chlorophyll to carotenoid content
TOL: Tolerance index
TSW: Thousand seed weight
UP: Number of umbels per plant
UU: Number of umbelets per umbel
YSI: Yield stability index
