# Supplementary material for: Genetic analysis and physiological relationships of drought response in fennel: Interaction with mating system
Source: PLoS One. 2022 Nov 29;17(11):e0277926. doi: 10.1371/journal.pone.0277926 (PMC9707804; doi:10.1371/journal.pone.0277926)
Supplement: S1 Table — (DOC) [file pone.0277926.s001.doc]

| **S1 Table -** Split-plot in time ANOVA for measured traits in 30 S1 families of fennel evaluated under two moisture conditions (normal and water deficit) during 2 years (2019- 2020). | | | | | | | | | | |
| --- | --- | --- | --- | --- | --- | --- | --- | --- | --- | --- |
| Traits | Moisture environment (E) (df = 1) | Replication / E  (df = 2) | Family (F)  (df = 29) | F × E  (df = 29) | F × Rep (E)  (df = 58) | Year (Y)  (df = 1) | E × Y  (df = 1) | F × Y  (df = 29) | E × F × Y  (df = 29) | Error  (df =58) |
| Days to germination, DG (day) | 31.54 ^*^ | 0.10 ^n.s^ | 32.05 ^**^ | 1.77 ^**^ | 0.36 ^n.s^ | 0.94 ^n.s^ | 18.70 ^**^ | 0.69 ^**^ | 0.42 ^n.s^ | 0.27 |
| Days to flowering, DF (day) | 592.20 ^*^ | 0.94 ^n.s^ | 317.79 ^**^ | 3.64 ^n.s^ | 4.07 ^*^ | 3912.34 ^**^ | 9.20 ^*^ | 20.47 ^**^ | 4.14 ^*^ | 2.28 |
| Days to maturity, DM (day) | 27030.04 ^**^ | 2.20 ^n.s^ | 270.38 ^**^ | 145.53 ^**^ | 4.80 ^n.s^ | 100327.70 ^**^ | 611.20 ^**^ | 118.92 ^**^ | 21.92 ^**^ | 4.56 |
| Plant height, PHT (cm) | 10436.59 ^*^ | 50.02 ^*^ | 666.32 ^**^ | 65.41 ^**^ | 28.34 ^**^ | 34.74 ^n.s^ | 105.91 ^**^ | 26.08 ^**^ | 27.17 ^**^ | 8.82 |
| Plant fresh weight, FW (g/plant) | 309647.64 ^**^ | 37.45 ^n.s^ | 12081.33 ^**^ | 6122.90 ^**^ | 140.64 ^**^ | 10956.07 ^**^ | 19.87 ^n.s^ | 648.09 ^**^ | 557.81 ^**^ | 58.20 |
| Plant dry weight, DW (g/plant) | 44379.79 ^**^ | 1.54 ^n.s^ | 782.78 ^**^ | 398.61 ^**^ | 2.52 ^n.s^ | 9821.06 ^**^ | 2827.90 ^**^ | 117.08 ^**^ | 68.55 ^**^ | 2.26 |
| Number of umbels per plant, UP | 2618.88 ^*^ | 1.67 ^n.s^ | 91.63 ^**^ | 17.93 ^**^ | 1.14 ^n.s^ | 68.48 ^**^ | 44.20 ^**^ | 8.05 ^**^ | 2.52 ^**^ | 0.80 |
| Number of umbelets per umbel, UU | 203.32 ^*^ | 0.48 ^n.s^ | 15.14 ^**^ | 2.36 ^n.s^ | 2.52 ^n.s^ | 1188.59 ^**^ | 151.85 ^**^ | 11.56 ^**^ | 2.21 ^n.s^ | 2.75 |
| Number of seeds per umbelets, SU | 1401.67 ^*^ | 0.56 ^n.s^ | 47.15 ^**^ | 12.63 ^*^ | 3.16 ^n.s^ | 5.64 ^n.s^ | 964.81 ^**^ | 21.71 ^**^ | 10.51 ^n.s^ | 6.92 |
| Seed yield per plant, SYP (g/plant) | 1698.78 ^**^ | 0.28 ^n.s^ | 75.76 ^**^ | 18.86 ^**^ | 1.41 ^**^ | 123.09 ^**^ | 41.08 ^**^ | 5.03 ^**^ | 2.60 ^**^ | 0.29 |
| Harvest index, HI (%) | 2676.81 ^*^ | 15.78 ^n.s^ | 857.22 ^**^ | 230.22 ^**^ | 21.95 ^n.s^ | 1783.02 ^**^ | 254.20 ^**^ | 79.27 ^**^ | 51.87 ^**^ | 14.41 |
| Thousand seed weight, TSW (g) | 24.7491 ^*^ | 0.0108 ^n.s^ | 0.7447 ^**^ | 0.1466 ^**^ | 0.0244 ^**^ | 0.2095 ^**^ | 0.0650 ^*^ | 0.0237 ^**^ | 0.0182 ^*^ | 0.0102 |
| Essential oil content, EOC (%) | 8.3179 ^*^ | 0.0191 ^n.s^ | 0.8329 ^**^ | 0.3096 ^**^ | 0.0098 ^n.s^ | 0.0437 ^*^ | 0.0112 ^n.s^ | 0.2405 ^**^ | 0.2451 ^**^ | 0.0073 |
| Seed length, SL (mm) | 32.4062 ^**^ | 0.0001 ^n.s^ | 1.4183 ^**^ | 0.4145 ^**^ | 0.0243 ^**^ | 6.1344 ^**^ | 0.3768 ^**^ | 0.1317 ^**^ | 0.0436 ^**^ | 0.0118 |
| Seed width, SW (mm) | 2.1679 ^*^ | 0.0032 ^n.s^ | 0.0686 ^**^ | 0.0164 ^**^ | 0.0020 ^**^ | 0.1777 ^**^ | 0.0033 ^n.s^ | 0.0043 ^**^ | 0.0026 ^**^ | 0.0008 |
| Relative water content, RWC (%) | 3754.96 ^*^ | 6.20 ^n.s^ | 65.87 ^**^ | 25.79 ^**^ | 3.23 ^*^ | 23.57 ^**^ | 39.58 ^**^ | 9.04 ^**^ | 5.05 ^**^ | 1.77 |
| Proline content , PRO (µmol g^-1^) | 0.18962 ^*^ | 0.00019 ^n.s^ | 0.00259 ^**^ | 0.00074 ^**^ | 0.00008 ^n.s^ | 0.03366 ^**^ | 0.01701 ^**^ | 0.00026 ^**^ | 0.00022 ^**^ | 0.00009 |
| Chlorophyll *a* content, Chl *a* (mg g ^-1^) | 64.648 ^*^ | 0.176 ^*^ | 2.178 ^**^ | 0.761 ^**^ | 0.068 ^*^ | 0.067 ^n.s^ | 0.358 ^**^ | 0.076 ^*^ | 0.068 ^*^ | 0.040 |
| Chlorophyll *b* content, Chl *b* (mg g ^-1^) | 4.4926 ^*^ | 0.0099 ^n.s^ | 0.1297 ^**^ | 0.0539 ^**^ | 0.0126 ^**^ | 0.0212 ^n.s^ | 0.0004 ^n.s^ | 0.0163 ^**^ | 0.0122 ^**^ | 0.0056 |
| Carotenoid content, CAR (mg g ^-1^) | 16.9622 ^*^ | 0.0532 ^*^ | 0.3525 ^**^ | 0.1068 ^**^ | 0.0208 ^**^ | 0.8038 ^**^ | 0.1565 ^**^ | 0.0152 ^*^ | 0.0168 ^**^ | 0.0081 |
| Total chlorophyll content, TChl (mg g ^-1^) | 103.2258 ^*^ | 0.1024 ^n.s^ | 3.0117 ^**^ | 0.7926 ^**^ | 0.0993 ^**^ | 0.1633 ^*^ | 0.3362 ^**^ | 0.1066 ^**^ | 0.0857 ^**^ | 0.0396 |
| Chl *a*/Chl *b* | 2037.3195 ^n.s^ | 35.9306 ^n.s^ | 133.6393 ^**^ | 145.1079 ^**^ | 16.0235 ^n.s^ | 29.9905 ^n.s^ | 11.3837 ^n.s^ | 24.7799 ^*^ | 32.7796 ^**^ | 14.7293 |
| Tchl/CAR | 7.6652 ^n.s^ | 0.2983 ^n.s^ | 5.7795 ^**^ | 5.4609 ^**^ | 0.2155 ^*^ | 2.9911 ^**^ | 2.5336 ^**^ | 0.5877 ^**^ | 0.4116 ^**^ | 0.1304 |
| * and ** show significance at the 0.05 and 0.01 probability levels, respectively.  n.s: not significant | | | | | | | | | | |
