# Supplementary material for: Genetic analysis and physiological relationships of drought response in fennel: Interaction with mating system
Source: PLoS One. 2022 Nov 29;17(11):e0277926. doi: 10.1371/journal.pone.0277926 (PMC9707804; doi:10.1371/journal.pone.0277926)
Supplement: S2 Table — (DOC) [file pone.0277926.s002.doc]

| **S2 Table -** Split-plot in time ANOVA for measured traits in 49 HS families (OP populations)of fennel evaluated under two moisture conditions (normal and water deficit) during 2 years (2019- 2020). | | | | | | | | | | |
| --- | --- | --- | --- | --- | --- | --- | --- | --- | --- | --- |
| Traits | Moisture environment (E) (df = 1) | Replication / E  (df = 2) | Family (F)  (df = 48) | F × E  (df = 48) | F × Rep (E)  (df = 96) | Year (Y)  (df = 1) | E × Y  (df = 1) | F × Y  (df = 48) | E × F × Y  (df = 48) | Error  (df =96) |
| Days to germination, DG (day) | 72.00 ^n.s^ | 4.50 ^n.s^ | 39.98 ^**^ | 2.30 ^n.s^ | 1.96 ^**^ | 771.68 ^**^ | 72.00 ^**^ | 5.63 ^**^ | 0.96 ^n.s^ | 1.21 |
| Days to flowering, DF (day) | 1454.15 ^*^ | 3.49 ^n.s^ | 317.41 ^**^ | 5.35 ^n.s^ | 4.98 ^n.s^ | 14365.13 ^**^ | 7.16 ^n.s^ | 16.85 ^**^ | 3.37 ^n.s^ | 4.80 |
| Days to maturity, DM (day) | 36972.57 ^*^ | 15.13 ^*^ | 283.83 ^**^ | 48.90 ^**^ | 4.53 ^n.s^ | 181331.02 ^**^ | 153.13 ^**^ | 26.02 ^**^ | 8.87 ^**^ | 3.55 |
| Plant height, PHT (cm) | 43391.42 ^*^ | 36.22 ^n.s^ | 1029.50 ^**^ | 302.19 ^**^ | 40.50 ^n.s^ | 2263.54 ^**^ | 35.08 ^n.s^ | 158.51 ^**^ | 83.59 ^**^ | 29.43 |
| Plant fresh weight, FW (g/plant) | 61300412.85 ^*^ | 150540.55 ^**^ | 284589.50 ^**^ | 163614.07^**^ | 5448.10 ^n.s^ | 7085031.02^**^ | 8881360.35^**^ | 60725.34 ^**^ | 53566.25 ^**^ | 5256.70 |
| Plant dry weight, DW (g/plant) | 655793.87 ^*^ | 198.09 ^n.s^ | 15654.99 ^**^ | 6699.25 ^**^ | 186.36 ^n.s^ | 60117.28 ^**^ | 30566.63 ^**^ | 4316.07 ^**^ | 2527.15 ^**^ | 254.54 |
| Number of umbels per plant, UP | 15539.58 ^*^ | 12.39 ^n.s^ | 161.37 ^**^ | 49.85 ^**^ | 11.44 ^n.s^ | 1959.83 ^**^ | 22.49 ^n.s^ | 39.05 ^**^ | 19.12 ^**^ | 8.55 |
| Number of umbelets per umbel, UU | 1542.90 ^*^ | 1.27 ^n.s^ | 19.90 ^**^ | 6.71 ^*^ | 4.57 ^n.s^ | 2068.62 ^**^ | 9.84 ^n.s^ | 7.40 ^**^ | 6.98 ^**^ | 3.62 |
| Number of seeds per umbelets, SU | 9171.42 ^*^ | 39.92 ^*^ | 55.10 ^**^ | 34.69 ^**^ | 12.62 ^n.s^ | 136.01 ^**^ | 118.69 ^**^ | 48.52 ^**^ | 24.36 ^**^ | 9.88 |
| Seed yield per plant, SYP (g/plant) | 30620.52 ^*^ | 43.98 ^*^ | 533.77 ^**^ | 145.64 ^**^ | 10.91 ^n.s^ | 404.72 ^**^ | 240.32 ^**^ | 59.70 ^**^ | 37.63 ^**^ | 8.05 |
| Harvest index, HI | 6950.46 ^**^ | 0.01 ^n.s^ | 1197.38 ^**^ | 280.43 ^**^ | 31.22 ^*^ | 319.23 ^**^ | 54.88 ^n.s^ | 235.41 ^**^ | 111.55 ^**^ | 21.56 |
| Thousand seed weight, TSW (g) | 66.3716 ^*^ | 0.0416 ^n.s^ | 2.0036 ^**^ | 0.1948 ^**^ | 0.0659 ^**^ | 0.7046 ^**^ | 0.0006 ^n.s^ | 0.2440 ^**^ | 0.0457 ^**^ | 0.0209 |
| Essential oil content, EOC (%) | 69.1740 ^**^ | 0.0010 ^n.s^ | 2.9068 ^**^ | 0.3008 ^**^ | 0.0564 ^n.s^ | 0.1891 ^*^ | 0.0002 ^n.s^ | 0.0856 ^**^ | 0.0684 ^*^ | 0.0444 |
| Seed length, SL (mm) | 87.3087 ^*^ | 0.0700 ^n.s^ | 2.2747 ^**^ | 0.3155 ^**^ | 0.0978 ^**^ | 0.2361 ^*^ | 0.0019 ^n.s^ | 0.3268 ^**^ | 0.0828 ^n.s^ | 0.0575 |
| Seed width, SW (mm) | 8.5905 ^**^ | 0.0024 ^n.s^ | 0.0849 ^**^ | 0.0832 ^**^ | 0.0076 ^*^ | 3.2604 ^**^ | 0.3171 ^**^ | 0.0218 ^**^ | 0.0170 ^**^ | 0.0050 |
| Relative water content, RWC (%) | 7621.80 ^*^ | 4.07 ^n.s^ | 125.44 ^**^ | 37.11 ^**^ | 5.54 ^n.s^ | 0.01 ^n.s^ | 13.37 ^n.s^ | 11.30 ^**^ | 5.14 ^n.s^ | 5.76 |
| Proline content , PRO (µmol g^-1^) | 0.01757 ^n.s^ | 0.00022 ^*^ | 0.00045 ^**^ | 0.00015 ^**^ | 0.00007 ^*^ | 0.05192 ^**^ | 0.00315 ^**^ | 0.00011 ^**^ | 0.00007 ^n.s^ | 0.00004 |
| Chlorophyll *a* content, Chl *a* (mg g ^-1^) | 191.726 ^*^ | 0.068 ^n.s^ | 2.377 ^**^ | 0.739 ^**^ | 0.146 ^n.s^ | 0.845 ^*^ | 0.119 ^n.s^ | 0.091 ^n.s^ | 0.067 ^n.s^ | 0.176 |
| Chlorophyll *b* content, Chl *b* (mg g ^-1^) | 12.1718 ^*^ | 0.0878 ^**^ | 0.1630 ^**^ | 0.0733 ^*^ | 0.0419 ^**^ | 0.6882 ^**^ | 0.3667 ^**^ | 0.0190 ^**^ | 0.0179 ^**^ | 0.0086 |
| Carotenoid content, CAR (mg g ^-1^) | 24.044 ^*^ | 0.029 ^n.s^ | 0.281 ^**^ | 0.085 ^**^ | 0.039 ^n.s^ | 0.162 ^*^ | 0.184 ^*^ | 0.019 ^n.s^ | 0.019 ^n.s^ | 0.030 |
| Total chlorophyll content, TChl (mg g ^-1^) | 300.513 ^*^ | 0.310 ^n.s^ | 3.168 ^**^ | 0.862 ^**^ | 0.190 ^n.s^ | 0.008 ^n.s^ | 0.903 ^*^ | 0.105 ^n.s^ | 0.084 ^n.s^ | 0.179 |
| Chl *a*/Chl *b* | 181.59 ^n.s^ | 5.86 ^n.s^ | 68.67 ^**^ | 56.09 ^**^ | 12.73 ^**^ | 196.74 ^**^ | 4.61 ^n.s^ | 5.58 ^n.s^ | 5.45 ^n.s^ | 4.72 |
| Tchl/CAR | 14.78 ^*^ | 0.04 ^n.s^ | 1.58 ^**^ | 0.86 ^**^ | 0.34 ^n.s^ | 0.13 ^n.s^ | 0.23 ^n.s^ | 0.27 ^n.s^ | 0.21 ^n.s^ | 0.28 |
| * and ** show significance at the 0.05 and 0.01 probability levels, respectively.  n.s: not significant | | | | | | | | | | |
