# Supplementary material for: Genetic analysis and physiological relationships of drought response in fennel: Interaction with mating system
Source: PLoS One. 2022 Nov 29;17(11):e0277926. doi: 10.1371/journal.pone.0277926 (PMC9707804; doi:10.1371/journal.pone.0277926)
Supplement: S3 Table — (DOC) [file pone.0277926.s003.doc]

| **S3 Table -** Mean comparisons of some important agro-morphological characters and essential oil content of S1 population of fennel during 2019-2020 under normal and water deficit conditions. | | | | | | | | | | | | | | |
| --- | --- | --- | --- | --- | --- | --- | --- | --- | --- | --- | --- | --- | --- | --- |
| Genotype | Days to flowering | |  | Seed yield (g/plant) | |  | Harvest index (%) | |  | Thousand seed weight (g) | |  | Essential oil content (%) | |
|  | Normal | Stress |  | Normal | Stress |  | Normal | Stress |  | Normal | Stress |  | Normal | Stress |
| S1-2 | 66.00 | 63.25 |  | 5.35 | 3.15 |  | 27.96 | 45.89 |  | 2.59 | 2.02 |  | 0.86 | 1.83 |
| S1-4 | 47.00 | 43.50 |  | 16.06 | 1.67 |  | 43.94 | 32.67 |  | 2.50 | 1.97 |  | 1.26 | 1.89 |
| S1-5 | 57.25 | 53.00 |  | 8.58 | 1.47 |  | 37.98 | 39.35 |  | 3.10 | 2.04 |  | 0.79 | 1.12 |
| S1-8 | 65.00 | 64.75 |  | 9.55 | 3.47 |  | 25.82 | 40.38 |  | 2.48 | 2.02 |  | 0.64 | 1.40 |
| S1-9 | 53.75 | 51.25 |  | 18.53 | 8.13 |  | 22.81 | 28.29 |  | 2.49 | 1.44 |  | 0.98 | 1.92 |
| S1-13 | 62.25 | 61.25 |  | 8.98 | 6.10 |  | 16.84 | 40.78 |  | 2.50 | 1.57 |  | 1.24 | 1.61 |
| S1-26 | 50.50 | 47.00 |  | 10.49 | 3.56 |  | 25.64 | 17.82 |  | 2.25 | 1.72 |  | 1.21 | 1.51 |
| S1-27 | 57.00 | 53.50 |  | 8.68 | 3.88 |  | 30.82 | 51.24 |  | 2.97 | 2.45 |  | 1.56 | 2.08 |
| S1-28 | 57.75 | 53.25 |  | 8.17 | 2.69 |  | 16.49 | 23.03 |  | 2.57 | 1.89 |  | 1.02 | 1.19 |
| S1-29 | 71.75 | 69.00 |  | 9.71 | 3.12 |  | 13.96 | 42.32 |  | 3.28 | 1.94 |  | 1.61 | 2.43 |
| S1-30 | 60.50 | 58.75 |  | 10.68 | 4.95 |  | 20.59 | 37.63 |  | 2.82 | 2.11 |  | 1.34 | 1.07 |
| S1-31 | 55.25 | 49.50 |  | 11.80 | 5.78 |  | 28.03 | 40.87 |  | 2.84 | 2.10 |  | 1.30 | 1.64 |
| S1-32 | 52.25 | 47.25 |  | 8.45 | 2.40 |  | 32.33 | 25.69 |  | 2.74 | 1.98 |  | 1.54 | 1.23 |
| S1-33 | 59.50 | 54.75 |  | 14.42 | 7.14 |  | 26.25 | 46.67 |  | 2.81 | 1.94 |  | 1.94 | 1.72 |
| S1-35 | 60.50 | 55.50 |  | 14.80 | 6.39 |  | 32.65 | 42.63 |  | 3.25 | 2.39 |  | 1.88 | 1.73 |
| S1-36 | 55.00 | 51.75 |  | 6.95 | 3.55 |  | 19.09 | 16.67 |  | 2.37 | 2.03 |  | 1.40 | 2.58 |
| S1-37 | 56.75 | 56.00 |  | 3.64 | 1.74 |  | 9.77 | 13.66 |  | 3.04 | 2.49 |  | 0.87 | 1.59 |
| S1-38 | 55.75 | 52.25 |  | 16.14 | 6.81 |  | 24.38 | 40.93 |  | 2.57 | 1.88 |  | 0.95 | 1.45 |
| S1-39 | 49.50 | 47.25 |  | 11.34 | 2.19 |  | 24.19 | 13.80 |  | 2.58 | 2.24 |  | 1.13 | 1.46 |
| S1-40 | 58.50 | 56.50 |  | 5.40 | 0.80 |  | 23.16 | 15.80 |  | 2.09 | 1.56 |  | 1.28 | 1.94 |
| S1-41 | 61.50 | 58.00 |  | 8.02 | 4.08 |  | 21.40 | 32.31 |  | 2.65 | 1.55 |  | 1.39 | 1.58 |
| S1-42 | 46.25 | 43.75 |  | 1.47 | 0.98 |  | 7.42 | 10.20 |  | 2.18 | 1.84 |  | 1.55 | 2.47 |
| S1-43 | 53.00 | 51.25 |  | 3.17 | 0.72 |  | 7.67 | 9.20 |  | 2.67 | 1.79 |  | 1.10 | 1.06 |
| S1-44 | 56.75 | 52.25 |  | 6.89 | 1.34 |  | 20.20 | 15.57 |  | 2.05 | 1.71 |  | 1.66 | 2.12 |
| S1-45 | 59.00 | 54.75 |  | 6.56 | 2.70 |  | 18.40 | 12.28 |  | 2.90 | 2.52 |  | 1.16 | 1.62 |
| S1-46 | 55.50 | 52.50 |  | 3.67 | 1.15 |  | 6.35 | 17.32 |  | 2.31 | 1.78 |  | 1.50 | 1.45 |
| S1-47 | 49.50 | 47.00 |  | 4.45 | 3.16 |  | 9.83 | 18.02 |  | 3.04 | 2.61 |  | 1.95 | 2.17 |
| S1-48 | 46.00 | 42.00 |  | 2.09 | 0.99 |  | 5.12 | 12.80 |  | 2.79 | 2.52 |  | 1.73 | 1.82 |
| S1-49 | 66.50 | 64.75 |  | 6.01 | 2.28 |  | 8.89 | 11.44 |  | 1.92 | 1.54 |  | 1.72 | 2.05 |
| S1-50 | 56.75 | 52.75 |  | 12.60 | 6.63 |  | 14.13 | 27.25 |  | 2.47 | 1.93 |  | 1.67 | 1.67 |
| Mean | 56.75 | 53.61 |  | 8.75 | 3.43 |  | 20.74 | 27.41 |  | 2.63 | 1.98 |  | 1.34 | 1.71 |
| LSD | 2.43 | 2.61 |  | 1.68 | 0.76 |  | 3.58 | 7.75 |  | 0.14 | 0.22 |  | 0.07 | 0.17 |
| LSD, least significant difference | | | | | | | | | | | | | | |
