# Supplementary material for: Genetic analysis and physiological relationships of drought response in fennel: Interaction with mating system
Source: PLoS One. 2022 Nov 29;17(11):e0277926. doi: 10.1371/journal.pone.0277926 (PMC9707804; doi:10.1371/journal.pone.0277926)
Supplement: S4 Table — (DOC) [file pone.0277926.s004.doc]

| **S4 Table -** Mean comparisons of some important agro-morphological characters and essential oil content of OP population of fennel during 2019-2020 under normal and water deficit conditions. | | | | | | | | | | | | | | |
| --- | --- | --- | --- | --- | --- | --- | --- | --- | --- | --- | --- | --- | --- | --- |
| Genotype | Days to flowering | |  | Seed yield (g/plant) | |  | Harvest index (%) | |  | Thousand seed weight (g) | |  | Essential oil content (%) | |
|  | Normal | Stress |  | Normal | Stress |  | Normal | Stress |  | Normal | Stress |  | Normal | Stress |
| OP-1 | 59.00 | 54.00 |  | 18.73 | 5.16 |  | 41.71 | 28.53 |  | 4.49 | 3.03 |  | 1.48 | 2.31 |
| OP-2 | 68.25 | 64.25 |  | 20.23 | 9.46 |  | 37.34 | 46.17 |  | 4.28 | 3.63 |  | 3.09 | 3.98 |
| OP-3 | 74.00 | 71.50 |  | 45.39 | 8.56 |  | 39.86 | 26.14 |  | 3.84 | 3.11 |  | 2.89 | 3.72 |
| OP-4 | 56.75 | 50.50 |  | 43.20 | 7.41 |  | 40.27 | 44.60 |  | 3.55 | 3.20 |  | 2.67 | 4.14 |
| OP-5 | 61.50 | 60.50 |  | 24.50 | 14.00 |  | 50.42 | 67.91 |  | 4.47 | 3.83 |  | 1.83 | 2.70 |
| OP-6 | 52.50 | 50.50 |  | 18.54 | 4.59 |  | 9.65 | 18.54 |  | 2.54 | 1.59 |  | 0.75 | 0.78 |
| OP-7 | 64.00 | 61.50 |  | 22.34 | 14.31 |  | 16.06 | 26.10 |  | 4.36 | 3.25 |  | 1.89 | 2.96 |
| OP-8 | 71.50 | 65.00 |  | 31.32 | 14.27 |  | 36.94 | 55.45 |  | 3.34 | 3.11 |  | 2.65 | 3.43 |
| OP-9 | 55.75 | 53.25 |  | 64.14 | 17.02 |  | 23.65 | 26.28 |  | 3.46 | 2.31 |  | 3.00 | 3.90 |
| OP-10 | 58.00 | 55.25 |  | 30.02 | 5.24 |  | 56.52 | 34.62 |  | 3.17 | 2.85 |  | 2.09 | 2.95 |
| OP-11 | 66.50 | 63.00 |  | 41.31 | 11.93 |  | 17.88 | 35.13 |  | 3.81 | 3.08 |  | 2.84 | 4.47 |
| OP-12 | 69.25 | 64.75 |  | 41.77 | 11.37 |  | 46.18 | 47.46 |  | 3.66 | 3.02 |  | 1.99 | 3.03 |
| OP-13 | 66.25 | 62.00 |  | 23.96 | 16.88 |  | 10.16 | 29.16 |  | 3.95 | 3.12 |  | 2.15 | 3.36 |
| OP-14 | 51.00 | 48.75 |  | 24.91 | 7.19 |  | 26.37 | 46.79 |  | 2.98 | 2.14 |  | 1.92 | 2.42 |
| OP-15 | 51.75 | 48.75 |  | 18.92 | 3.09 |  | 42.69 | 43.52 |  | 3.86 | 2.47 |  | 2.31 | 3.80 |
| OP-16 | 57.50 | 54.25 |  | 27.13 | 4.65 |  | 41.25 | 26.29 |  | 3.66 | 2.50 |  | 1.91 | 2.17 |
| OP-17 | 59.50 | 57.25 |  | 23.20 | 6.87 |  | 64.59 | 57.71 |  | 3.32 | 2.26 |  | 2.22 | 3.80 |
| OP-18 | 66.25 | 63.50 |  | 23.61 | 10.29 |  | 28.25 | 54.70 |  | 4.10 | 2.93 |  | 2.22 | 3.38 |
| OP-19 | 63.75 | 61.50 |  | 22.27 | 8.08 |  | 26.39 | 30.28 |  | 3.73 | 2.97 |  | 2.79 | 4.06 |
| OP-20 | 65.50 | 60.75 |  | 27.68 | 17.60 |  | 54.21 | 62.90 |  | 4.13 | 3.25 |  | 1.75 | 2.73 |
| OP-21 | 56.75 | 54.50 |  | 31.56 | 9.34 |  | 52.53 | 59.22 |  | 3.39 | 2.74 |  | 2.63 | 3.35 |
| OP-22 | 59.00 | 55.75 |  | 45.03 | 10.22 |  | 37.27 | 39.00 |  | 4.15 | 2.73 |  | 1.52 | 3.17 |
| OP-23 | 72.00 | 66.75 |  | 30.87 | 10.54 |  | 18.63 | 30.65 |  | 3.73 | 2.52 |  | 2.16 | 3.20 |
| OP-24 | 58.25 | 52.25 |  | 16.85 | 6.00 |  | 35.51 | 39.53 |  | 4.35 | 3.53 |  | 1.38 | 2.38 |
| OP-25 | 65.25 | 60.00 |  | 27.51 | 6.12 |  | 51.44 | 41.81 |  | 4.47 | 2.80 |  | 1.68 | 2.48 |
| OP-26 | 70.75 | 65.50 |  | 51.43 | 31.67 |  | 30.99 | 46.85 |  | 4.15 | 3.56 |  | 2.05 | 2.62 |
| OP-27 | 70.75 | 68.00 |  | 41.70 | 14.79 |  | 48.89 | 49.77 |  | 4.50 | 3.68 |  | 2.59 | 3.37 |
| OP-28 | 69.50 | 66.75 |  | 25.85 | 9.72 |  | 28.89 | 44.74 |  | 4.26 | 3.13 |  | 2.45 | 3.38 |
| OP-30 | 64.00 | 61.75 |  | 26.50 | 17.65 |  | 19.14 | 29.44 |  | 3.86 | 3.28 |  | 1.81 | 2.30 |
| OP-31 | 71.00 | 67.25 |  | 26.23 | 18.02 |  | 13.96 | 30.30 |  | 4.69 | 4.13 |  | 2.44 | 2.80 |
| OP-32 | 64.25 | 60.25 |  | 44.36 | 23.67 |  | 19.08 | 57.57 |  | 3.90 | 3.12 |  | 2.54 | 2.73 |
| OP-33 | 74.75 | 72.50 |  | 42.24 | 24.52 |  | 30.69 | 37.78 |  | 5.23 | 4.19 |  | 3.50 | 4.07 |
| OP-34 | 74.00 | 69.00 |  | 24.89 | 10.27 |  | 28.67 | 47.50 |  | 3.46 | 2.87 |  | 3.02 | 3.77 |
| OP-35 | 74.00 | 70.50 |  | 39.05 | 20.96 |  | 22.09 | 31.77 |  | 4.97 | 4.15 |  | 2.69 | 3.26 |
| OP-36 | 65.75 | 62.25 |  | 19.73 | 10.18 |  | 30.63 | 24.59 |  | 3.59 | 3.20 |  | 3.50 | 3.94 |
| OP-37 | 61.25 | 55.75 |  | 25.66 | 10.90 |  | 12.66 | 20.48 |  | 4.49 | 3.81 |  | 1.97 | 2.80 |
| OP-38 | 68.25 | 60.75 |  | 60.28 | 36.35 |  | 25.23 | 31.83 |  | 3.75 | 3.33 |  | 2.29 | 3.45 |
| OP-39 | 59.75 | 57.25 |  | 39.17 | 22.35 |  | 20.54 | 32.30 |  | 4.13 | 3.62 |  | 2.16 | 2.62 |
| OP-40 | 72.75 | 68.25 |  | 38.15 | 17.25 |  | 29.12 | 43.45 |  | 3.64 | 3.00 |  | 3.03 | 3.51 |
| OP-41 | 73.25 | 70.75 |  | 42.32 | 26.83 |  | 39.83 | 37.61 |  | 4.19 | 3.30 |  | 2.80 | 3.96 |
| OP-42 | 66.25 | 65.25 |  | 35.03 | 16.45 |  | 31.78 | 53.08 |  | 3.71 | 2.58 |  | 3.48 | 4.14 |
| OP-43 | 72.50 | 65.50 |  | 20.61 | 9.59 |  | 12.34 | 19.60 |  | 3.69 | 3.21 |  | 1.98 | 3.50 |
| OP-44 | 67.75 | 59.75 |  | 27.30 | 16.41 |  | 24.90 | 48.50 |  | 3.36 | 2.73 |  | 3.24 | 3.78 |
| OP-45 | 65.75 | 60.25 |  | 22.74 | 10.26 |  | 8.05 | 19.43 |  | 4.46 | 3.60 |  | 2.38 | 3.26 |
| OP-46 | 61.25 | 57.25 |  | 23.86 | 11.37 |  | 15.09 | 19.05 |  | 3.94 | 3.05 |  | 2.18 | 3.14 |
| OP-47 | 68.50 | 63.25 |  | 27.23 | 16.46 |  | 15.64 | 39.61 |  | 4.71 | 3.84 |  | 3.22 | 3.42 |
| OP-48 | 63.00 | 59.00 |  | 24.93 | 15.54 |  | 13.74 | 40.80 |  | 4.04 | 3.47 |  | 2.69 | 3.31 |
| OP-49 | 75.00 | 72.50 |  | 22.93 | 13.26 |  | 13.92 | 20.27 |  | 3.39 | 2.78 |  | 2.94 | 3.57 |
| OP-50 | 62.75 | 58.50 |  | 35.61 | 22.01 |  | 12.75 | 22.26 |  | 3.94 | 2.97 |  | 2.26 | 2.80 |
| Mean | 65.03 | 61.18 |  | 31.28 | 13.60 |  | 29.68 | 38.10 |  | 3.93 | 3.11 |  | 2.39 | 3.23 |
| LSD | 3.28 | 2.91 |  | 5.31 | 3.03 |  | 7.13 | 7.29 |  | 0.34 | 0.23 |  | 0.32 | 0.30 |
| LSD, least significant difference | | | | | | | | | | | | | | |
