# Supplementary material for: Genetic analysis and physiological relationships of drought response in fennel: Interaction with mating system
Source: PLoS One. 2022 Nov 29;17(11):e0277926. doi: 10.1371/journal.pone.0277926 (PMC9707804; doi:10.1371/journal.pone.0277926)
Supplement: S5 Table — (DOC) [file pone.0277926.s005.doc]

| **S5 Table -** General combining ability (GCA) of some important agro-morphological characters and essential oil content of OP population of fennel during 2019-2020 under normal and water deficit conditions. | | | | | | | | | | | | | | |
| --- | --- | --- | --- | --- | --- | --- | --- | --- | --- | --- | --- | --- | --- | --- |
| Genotype | Days to flowering | |  | Seed yield per plant (g per plant) | |  | Harvest index (%) | |  | Thousand seed weight (g) | |  | Essential oil content (%) | |
|  | Normal | Stress |  | Normal | Stress |  | Normal | Stress |  | Normal | Stress |  | Normal | Stress |
| OP-1 | -6.03 | -7.18 |  | -12.55 | -8.44 |  | 12.03 | -9.57 |  | 0.55 | -0.08 |  | -0.91 | -0.92 |
| OP-2 | 3.22 | 3.07 |  | -11.05 | -4.14 |  | 7.66 | 8.07 |  | 0.34 | 0.52 |  | 0.70 | 0.75 |
| OP-3 | 8.97 | 10.32 |  | 14.10 | -5.04 |  | 10.18 | -11.96 |  | -0.09 | -0.01 |  | 0.50 | 0.49 |
| OP-4 | -8.28 | -10.68 |  | 11.91 | -6.19 |  | 10.59 | 6.50 |  | -0.38 | 0.08 |  | 0.29 | 0.91 |
| OP-5 | -3.53 | -0.68 |  | -6.78 | 0.39 |  | 20.74 | 29.80 |  | 0.54 | 0.71 |  | -0.56 | -0.53 |
| OP-6 | -12.53 | -10.68 |  | -12.75 | -9.01 |  | -20.04 | -19.57 |  | -1.39 | -1.53 |  | -1.64 | -2.45 |
| OP-7 | -1.03 | 0.32 |  | -8.94 | 0.70 |  | -13.63 | -12.00 |  | 0.43 | 0.14 |  | -0.50 | -0.27 |
| OP-8 | 6.47 | 3.82 |  | 0.04 | 0.66 |  | 7.26 | 17.35 |  | -0.59 | 0.00 |  | 0.26 | 0.21 |
| OP-9 | -9.28 | -7.93 |  | 32.86 | 3.41 |  | -6.03 | -11.82 |  | -0.47 | -0.80 |  | 0.61 | 0.67 |
| OP-10 | -7.03 | -5.93 |  | -1.26 | -8.37 |  | 26.84 | -3.48 |  | -0.77 | -0.27 |  | -0.30 | -0.28 |
| OP-11 | 1.47 | 1.82 |  | 10.03 | -1.68 |  | -11.80 | -2.97 |  | -0.12 | -0.03 |  | 0.45 | 1.24 |
| OP-12 | 4.22 | 3.57 |  | 10.48 | -2.24 |  | 16.49 | 9.36 |  | -0.27 | -0.10 |  | -0.40 | -0.19 |
| OP-13 | 1.22 | 0.82 |  | -7.32 | 3.27 |  | -19.52 | -8.94 |  | 0.02 | 0.01 |  | -0.23 | 0.14 |
| OP-14 | -14.03 | -12.43 |  | -6.37 | -6.41 |  | -3.31 | 8.69 |  | -0.96 | -0.97 |  | -0.47 | -0.81 |
| OP-15 | -13.28 | -12.43 |  | -12.36 | -10.52 |  | 13.01 | 5.41 |  | -0.07 | -0.64 |  | -0.07 | 0.58 |
| OP-16 | -7.53 | -6.93 |  | -4.15 | -8.95 |  | 11.56 | -11.82 |  | -0.28 | -0.62 |  | -0.48 | -1.06 |
| OP-17 | -5.53 | -3.93 |  | -8.08 | -6.74 |  | 34.91 | 19.61 |  | -0.62 | -0.85 |  | -0.17 | 0.58 |
| OP-18 | 1.22 | 2.32 |  | -7.67 | -3.32 |  | -1.43 | 16.60 |  | 0.16 | -0.19 |  | -0.16 | 0.15 |
| OP-19 | -1.28 | 0.32 |  | -9.02 | -5.52 |  | -3.29 | -7.82 |  | -0.20 | -0.14 |  | 0.40 | 0.83 |
| OP-20 | 0.47 | -0.43 |  | -3.60 | 3.99 |  | 24.53 | 24.79 |  | 0.20 | 0.14 |  | -0.63 | -0.50 |
| OP-21 | -8.28 | -6.68 |  | 0.28 | -4.27 |  | 22.84 | 21.12 |  | -0.55 | -0.37 |  | 0.24 | 0.12 |
| OP-22 | -6.03 | -5.43 |  | 13.75 | -3.39 |  | 7.59 | 0.90 |  | 0.22 | -0.39 |  | -0.87 | -0.06 |
| OP-23 | 6.97 | 5.57 |  | -0.41 | -3.07 |  | -11.06 | -7.45 |  | -0.20 | -0.59 |  | -0.23 | -0.03 |
| OP-24 | -6.78 | -8.93 |  | -14.43 | -7.60 |  | 5.83 | 1.42 |  | 0.41 | 0.42 |  | -1.01 | -0.84 |
| OP-25 | 0.22 | -1.18 |  | -3.77 | -7.48 |  | 21.75 | 3.71 |  | 0.53 | -0.31 |  | -0.70 | -0.75 |
| OP-26 | 5.72 | 4.32 |  | 20.15 | 18.07 |  | 1.31 | 8.75 |  | 0.21 | 0.45 |  | -0.34 | -0.61 |
| OP-27 | 5.72 | 6.82 |  | 10.41 | 1.18 |  | 19.21 | 11.67 |  | 0.57 | 0.56 |  | 0.20 | 0.14 |
| OP-28 | 4.47 | 5.57 |  | -5.44 | -3.89 |  | -0.79 | 6.64 |  | 0.32 | 0.02 |  | 0.06 | 0.16 |
| OP-30 | -1.03 | 0.57 |  | -4.78 | 4.05 |  | -10.54 | -8.66 |  | -0.07 | 0.17 |  | -0.58 | -0.92 |
| OP-31 | 5.97 | 6.07 |  | -5.05 | 4.42 |  | -15.72 | -7.81 |  | 0.76 | 1.02 |  | 0.05 | -0.43 |
| OP-32 | -0.78 | -0.93 |  | 13.08 | 10.06 |  | -10.60 | 19.46 |  | -0.03 | 0.01 |  | 0.15 | -0.49 |
| OP-33 | 9.72 | 11.32 |  | 10.96 | 10.91 |  | 1.01 | -0.32 |  | 1.29 | 1.08 |  | 1.11 | 0.84 |
| OP-34 | 8.97 | 7.82 |  | -6.40 | -3.33 |  | -1.01 | 9.39 |  | -0.47 | -0.24 |  | 0.63 | 0.54 |
| OP-35 | 8.97 | 9.32 |  | 7.77 | 7.35 |  | -7.59 | -6.33 |  | 1.04 | 1.04 |  | 0.31 | 0.03 |
| OP-36 | 0.72 | 1.07 |  | -11.55 | -3.42 |  | 0.95 | -13.51 |  | -0.34 | 0.09 |  | 1.11 | 0.71 |
| OP-37 | -3.78 | -5.43 |  | -5.63 | -2.71 |  | -17.02 | -17.62 |  | 0.56 | 0.70 |  | -0.41 | -0.43 |
| OP-38 | 3.22 | -0.43 |  | 29.00 | 22.75 |  | -4.46 | -6.27 |  | -0.18 | 0.22 |  | -0.10 | 0.23 |
| OP-39 | -5.28 | -3.93 |  | 7.89 | 8.75 |  | -9.15 | -5.81 |  | 0.20 | 0.50 |  | -0.23 | -0.61 |
| OP-40 | 7.72 | 7.07 |  | 6.87 | 3.64 |  | -0.56 | 5.35 |  | -0.30 | -0.12 |  | 0.64 | 0.29 |
| OP-41 | 8.22 | 9.57 |  | 11.04 | 13.23 |  | 10.15 | -0.49 |  | 0.25 | 0.19 |  | 0.41 | 0.73 |
| OP-42 | 1.22 | 4.07 |  | 3.75 | 2.85 |  | 2.10 | 14.97 |  | -0.23 | -0.53 |  | 1.09 | 0.91 |
| OP-43 | 7.47 | 4.32 |  | -10.67 | -4.01 |  | -17.35 | -18.50 |  | -0.25 | 0.09 |  | -0.40 | 0.27 |
| OP-44 | 2.72 | -1.43 |  | -3.98 | 2.80 |  | -4.78 | 10.40 |  | -0.57 | -0.38 |  | 0.85 | 0.55 |
| OP-45 | 0.72 | -0.93 |  | -8.55 | -3.35 |  | -21.64 | -18.67 |  | 0.53 | 0.49 |  | -0.01 | 0.03 |
| OP-46 | -3.78 | -3.93 |  | -7.43 | -2.24 |  | -14.59 | -19.05 |  | 0.01 | -0.06 |  | -0.21 | -0.09 |
| OP-47 | 3.47 | 2.07 |  | -4.05 | 2.86 |  | -14.04 | 1.50 |  | 0.77 | 0.73 |  | 0.83 | 0.19 |
| OP-48 | -2.03 | -2.18 |  | -6.35 | 1.94 |  | -15.94 | 2.69 |  | 0.11 | 0.36 |  | 0.30 | 0.09 |
| OP-49 | 9.97 | 11.32 |  | -8.35 | -0.34 |  | -15.77 | -17.84 |  | -0.55 | -0.33 |  | 0.55 | 0.35 |
| OP-50 | -2.28 | -2.68 |  | 4.33 | 8.40 |  | -16.93 | -15.84 |  | 0.01 | -0.15 |  | -0.13 | -0.43 |
| LSD | 3.28 | 2.91 |  | 5.31 | 3.03 |  | 7.13 | 7.29 |  | 0.34 | 0.23 |  | 0.32 | 0.30 |
| LSD, least significant difference | | | | | | | | | | | | | | |
