# Supplementary material for: Genetic analysis and physiological relationships of drought response in fennel: Interaction with mating system
Source: PLoS One. 2022 Nov 29;17(11):e0277926. doi: 10.1371/journal.pone.0277926 (PMC9707804; doi:10.1371/journal.pone.0277926)
Supplement: S6 Table — (DOC) [file pone.0277926.s006.doc]

| **S6 Table -** Heritability estimates of agro-morphological characters and essential oil content based on parent-offspring regression (h^2^_po_) in two populations of fennel. | | | | | |
| --- | --- | --- | --- | --- | --- |
| Traits | Selfed population (S1) | |  | Open-pollinated population (OP) | |
|  | Normal | Stress |  | Normal | Stress |
| DF | 75.58 | 79.16 |  | 71.34 | 74.24 |
| DM | 53.69 | 66.01 |  | 72.13 | 79.97 |
| PHT | 77.53 | 79.41 |  | 68.42 | 78.14 |
| FW | 73.13 | 78.22 |  | 61.72 | 67.58 |
| DW | 73.75 | 75.09 |  | 64.64 | 77.72 |
| UP | 71.58 | 75.77 |  | 57.82 | 66.09 |
| UU | 39.12 | 41.04 |  | 40.66 | 48.18 |
| SU | 60.39 | 77.38 |  | 40.74 | 44.12 |
| SYP | 54.19 | 62.73 |  | 72.36 | 70.99 |
| HI | 78.28 | 71.73 |  | 75.55 | 61.35 |
| TSW | 76.78 | 70.63 |  | 71.66 | 73.76 |
| EOC | 77.94 | 78.01 |  | 78.88 | 77.51 |
| DF, Days to flowering; DM, Days to maturity; DW, Plant dry weight; EOC, Essential oil content; FW, Plant fresh weight; HI, Harvest index; PHT, Plant height; SU, Number of seeds per umbelets; SYP, Seed yield per plant; TSW, Thousand seed weight; UP, Number of umbels per plant; UU, Number of umbelets per umbel. | | | | | |
